# TGF-β3-expressing CD4^+^CD25^−^LAG3^+^ regulatory T cells control humoral immune responses

**DOI:** 10.1038/ncomms7329

**Published:** 2015-02-19

**Authors:** Tomohisa Okamura, Shuji Sumitomo, Kaoru Morita, Yukiko Iwasaki, Mariko Inoue, Shinichiro Nakachi, Toshihiko Komai, Hirofumi Shoda, Jun-ichi Miyazaki, Keishi Fujio, Kazuhiko Yamamoto

**Affiliations:** 1 Department of Allergy and Rheumatology, Graduate School of Medicine, The University of Tokyo, 7-3-1 Hongo, Bunkyo-ku, Tokyo 113-8655, Japan; 2 Max Planck-The University of Tokyo Center for Integrative Inflammology, The University of Tokyo, 4-6-1 Komaba, Meguro-ku, Tokyo 153-8505, Japan; 3 Division of Stem Cell Regulation Research, Osaka University Graduate School of Medicine, 2-2 Yamadaoka, Suita, Osaka 565-0871, Japan

## Abstract

Autoantibodies induce various autoimmune diseases, including systemic lupus erythematosus (SLE). We previously described that CD4^+^CD25^−^LAG3^+^ regulatory T cells (LAG3^+^ Treg) are regulated by Egr2, a zinc-finger transcription factor required for the induction of T-cell anergy. We herein demonstrate that LAG3^+^ Treg produce high amounts of TGF-β3 in an Egr2- and Fas-dependent manner. LAG3^+^ Treg require TGF-β3 to suppress B-cell responses in a murine model of lupus. Moreover, TGF-β3- and LAG3^+^ Treg-mediated suppression requires PD-1 expression on B cells. We also show that TGF-β3-expressing human LAG3^+^ Treg suppress antibody production and that SLE patients exhibit decreased frequencies of LAG3^+^ Treg. These results clarify the mechanism of B-cell regulation and suggest therapeutic strategies.

## Abstract (web summary)

B cells reactive against self antigens can cause autoimmune disease, but are normally suppressed by regulatory T cells (Tregs). Here the authors show that a subset of Tregs can suppress lupus in a mouse model by making TGF-β3 cytokine and by engaging an inhibitory PD-1 receptor on B cells.

## Graphical abstract

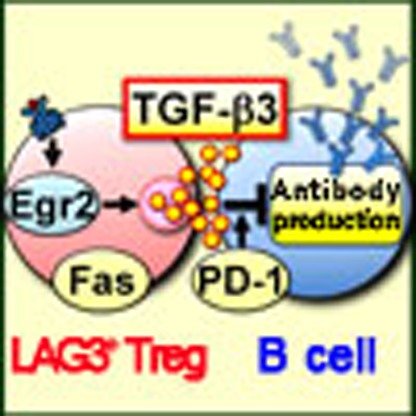

Autoantibodies induce various autoimmune diseases, including systemic lupus erythematosus (SLE)^1^, which is characterized by severe inflammation in multiple organ systems. The high-affinity autoantibodies primarily originating from the self-reactive B cells underwent somatic hypermutation in the germinal centre (GC)^2^. Follicular helper T (T_FH_) cells expressing CXCR5 have emerged as a lineage of helper T cells (Th cells) that are functionally specialized to provide help to B cells, allowing the formation of GC and the subsequent long-lived plasma cell differentiation. Therefore, regulation of the quality and quantity of T_FH_ cells and memory B-cell populations in GC (GCB) is important to prevent immunopathology. CD4^+^CD25^+^ Treg (CD25^+^ Treg) that express Foxp3 play the key roles in the maintenance of self-tolerance and suppress the activation of conventional T cells and dendritic cells^3^. Moreover, accumulating evidence indicates the essential role of CD25^+^ Treg, including CD4^+^CD25^+^CXCR5^+^ follicular Treg^2^ and CD4^+^CD25^+^CD69^−^ Treg^4^, in the regulation of humoral immunity. These observations highlight the protective role of CD25^+^ Treg in systemic autoimmunity; however, the disease induced by the absence of functional CD25^+^ Treg is quite different from SLE^1^,^5^. Moreover, a role for CD25^+^ Treg in SLE has not been clearly established^6^. Recent advances in understanding of CD8^+^ Treg have underscored the importance of Qa-1-restricted CD8^+^ Treg for the maintenance of B-cell tolerance. Mice with functional impairment in CD8^+^ Treg exhibit a lupus-like disease with a significant increase in T_FH_^7^. The development of systemic autoimmunity in B6.*Yaa* mutant mice is associated with a pronounced defect in CD8^+^ Treg activity^8^. Nevertheless, the actual contribution of CD8^+^ Treg to the regulation of human autoimmunity remains unclear.

Early growth response gene 2 (Egr2), a zinc-finger transcription factor, plays a critical role in hindbrain development and myelination of the peripheral nervous system^9^. In T cells, Egr2 is important for the maintenance of T-cell anergy by negatively regulating T-cell activation^10^. The involvement of Egr2 in the control of systemic autoimmunity was first suggested by the observation that lymphocyte-specific Egr2-deficient mice develop a lupus-like disease with no impact on the development of Foxp3-expressing CD25^+^ Treg^11^. Moreover, mice deficient for both Egr2 and Egr3 in B and T cells present lethal and early-onset systemic autoimmunity, suggesting a synergistic role for Egr2 and Egr3 in controlling B-cell tolerance^12^. We and our collaborators have shown that polymorphisms in *EGR2* influence SLE susceptibility in humans^13^. We have previously identified Egr2-controlled CD4^+^CD25^−^LAG3^+^ Treg (LAG3^+^ Treg)^14^. LAG3 is a CD4-related molecule that binds to MHC class II, and the binding induces immunoreceptor tyrosine-based activation motif (ITAM)-mediated inhibitory signalling^15^. Approximately 2% of the CD4^+^CD25^−^ T-cell population in the spleen express LAG3. These LAG3^+^ Treg produce high levels of interleukin (IL)-10 and are suppressive in a murine model of colitis in an IL-10-dependent manner. Unlike CD25^+^ Treg, high-affinity interactions with selecting peptide/MHC ligands expressed in the thymus do not induce the development of LAG3^+^ Treg. Recently, Gagliani *et al*.^16^ reported that concomitant expression of LAG3 and CD49b is specific for IL-10-producing type 1 T regulatory (Tr1) cells, confirming that LAG3 is one of the phenotypic markers of IL-10-producing Foxp3-independent CD4^+^ Treg.

The association between Egr2 and autoantibody-mediated systemic autoimmunity suggested a linkage between Egr2-expressing LAG3^+^ Treg and the control of B-cell responses. We herein demonstrate that LAG3^+^ Treg produce high amounts of transforming growth factor-β3 (TGF-β3) and suppress B-cell development and antibody production. In MRL/*lpr* lupus-prone mice, adoptive transfer of LAG3^+^ Treg from MRL/+ mice suppresses the progression of lupus in a TGF-β3-dependent manner. Expression of both Fas and Egr2 by LAG3^+^ Treg is necessary for TGF-β3 production and for the suppression of humoral immunity. These results clarify the mechanisms underlying LAG3^+^ Treg-mediated B-cell regulation.

## Results

### Egr2 mediates control of humoral immunity by LAG3^+^ Treg

**Figure 1:**
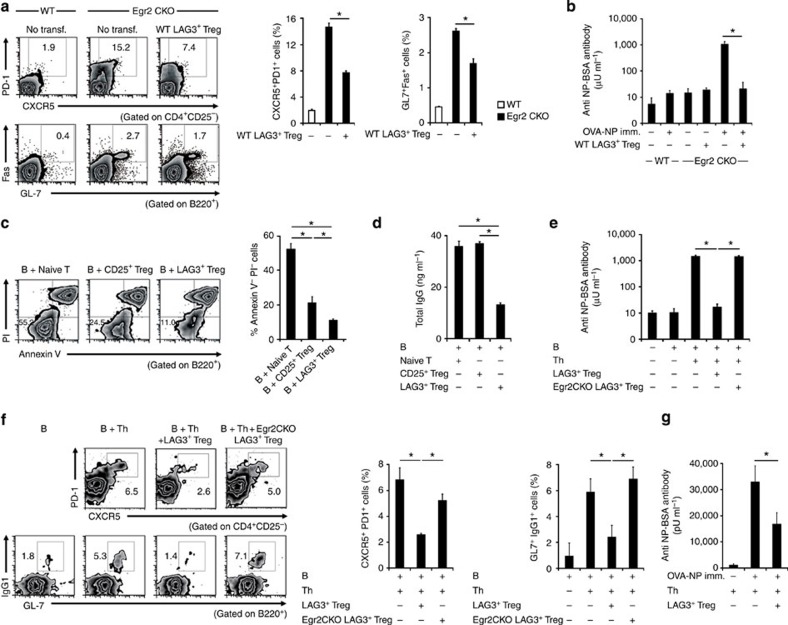
LAG3^+^ Treg exhibit Egr2-dependent control of antibody production. (**a**) Flow cytometry plots and quantification of CD4^+^CD25^−^CXCR5^+^PD-1^+^ T_FH_ and B220^+^GL-7^+^Fas^+^ GCB from WT or Egr2 CKO mice at 7 days with or without the adoptive transfer of WT LAG3^+^ Treg (*n*=5 per group). *P<0.05 (unpaired two-tailed Student’s *t*-test). (**b**) NP-specific antibody responses of WT and Egr2 CKO mice immunized once with 100 μg NP-OVA/alum with or without adoptive transfer of WT LAG3^+^ Treg. The serum levels of anti-NP-BSA antibodies were analysed with ELISA 7 days after immunization. See also Supplementary Fig. 1a (*n*=6 per group). *P<0.05 (Bonferroni post-test). (**c**,**d**) *In vitro* B-cell suppression by LAG3^+^ Treg. Each T-cell subset stimulated with anti-CD3 mAb was co-cultured with stimulated B cells. (**c**) Live B220^+^ B cells were quantified with AnnexinV/PI staining 72 h after anti-IgM stimulation (*n*=3 per group). *P<0.05 (Bonferroni post-test). (**d**) Total IgG was determined in anti-CD40/IL-4-stimulated B-cell culture supernatants on day 7 by ELISA (*n*=3 per group). *P<0.05 (Bonferroni post-test). (**e**) *In vivo* NP-specific antibody responses. C57BL/6 (B6) B cells and OT-II CD4^+^CD25^−^LAG3^−^ Th cells were injected into Rag1KO mice in combination with or without LAG3^+^ Treg from B6 mice 1 day before the immunization with NP-OVA/alum, and given a booster immunization 14 days after the primary immunization. Anti-NP-BSA antibodies in sera were analysed with ELISA 7 days after the booster immunization. See also Supplementary Fig. 1b (*n*=6 per group). *P<0.05 (Bonferroni post-test). (**f**) Flow cytometry plots and quantification of splenic T_FH_ and B220^+^GL7^+^IgG1^+^ GCB from the same mice as in **e**. *P<0.05 (Bonferroni post-test). (**g**) B-cell suppression by LAG3^+^ Treg in non-lymphopenic TEa mice. LAG3^+^ Treg from B6 mice and OT-II Th cells were injected into TEa mice and subsequently immunized with NP-OVA/alum once. Anti-NP-BSA antibody levels were determined with ELISA. See also Supplementary Fig. 1d (*n*=6 per group). *P<0.05 (post-test). Data are representative of three independent experiments. The means±s.d. are indicated.

To clarify the role of Egr2 in T cells, we generated T-cell-specific Egr2 conditional knockout (CKO) mice (*Egr2*^*fl*/*fl*^ CD4-*Cre*^+^). Egr2 CKO mice showed significant increases in the proportion of CD4^+^CD25^−^CXCR5^+^PD-1^+^ T_FH_ and B220^+^GL-7^+^Fas^+^ GCB (Fig. 1a), and they demonstrated enhanced 4-hydroxy-3-nitrophenylacetyl (NP)-specific antibody production following a single immunization with NP-ovalbumin (NP-OVA; Fig. 1b and Supplementary Fig. 1a). Transfer of wild-type (WT) LAG3^+^ Treg significantly suppressed the spontaneous differentiation of T_FH_ and GCB (Fig.1a) and inhibited excessive antibody production (Fig. 1b), indicating that LAG3^+^ Treg are able to suppress B-cell responses *in vivo*. To examine whether physiological number of WT LAG3^+^ Treg could improve excessive T_FH_ and GCB development in Egr2 CKO mice, we reconstituted Thy1.2^+^ Egr2 CKO-recipient mice with an equal number of bone marrow (BM) cells derived from Thy1.2^+^ Egr2 CKO mice and Thy1.1^+^ WT mice (Supplementary Fig. 2). Six weeks after the reconstitution, although less than 50% of splenocytes were derived from Egr2-sufficient WT mice, the excessive development of T_FH_ and GCB cells was significantly reduced (Supplementary Fig. 2b,c). This result suggested that the immunological abnormality of Egr2 CKO mice is dependent, at least in part, on the defects in Egr2-expressing LAG3^+^ Treg. In an *in vitro* T-cell/B-cell co-culture system, anti-CD3-stimulated WT LAG3^+^ Treg more efficiently reduced the percentage of viable anti-IgM-stimulated B cells as well as total IgG production from anti-CD40/IL-4-stimulated B cells when compared with CD25^+^ Treg (Fig. 1c,d). To evaluate B-cell responses *in vivo*, recombination-activating gene 1-deficient (Rag1KO) mice were transferred with WT B cells and CD4^+^CD25^−^LAG3^−^ helper T cells (Th cells) from OVA-specific OT-II T cell receptor (TCR) transgenic mice, and then immunized with NP-OVA twice. Strikingly, co-transfer of WT LAG3^+^ Treg effectively suppressed NP-specific antibody responses and the development of T_FH_ and GCB (Fig. 1e,f and Supplementary Fig. 1b). The enhanced GCB development and antibody production in Egr2 CKO mice suggested a pivotal role for Egr2 in B-cell regulation. Although Egr2 confers the phenotype of LAG3^+^ Treg^14^, T cells with the phenotype of LAG3^+^ Treg were also observed in the spleen of Egr2 CKO mice (Supplementary Fig. 1c). This result suggested that the expression of LAG3, the marker for LAG3^+^ Treg, is not fully dependent on Egr2. Egr2-deficient LAG3^+^ Treg (Supplementary Fig. 1c) failed to suppress *in vivo* B-cell antibody production and the development of T_FH_ and GCB (Fig. 1e,f). Thus, the expression of Egr2 on LAG3^+^ Treg is necessary for the suppression of B-cell responses. In transgenic mice that express green fluorescent protein (GFP) under the control of the Egr2 promoter (Egr2-GFP mice; Supplementary Fig. 3a), the expression of GFP in CD4^+^ T cells correlated with Egr2 protein expression (Supplementary Fig. 3b). The importance of Egr2 was confirmed by the observation that CD4^+^CD25^−^Egr2-GFP^+^ cells from Egr2-GFP mice also exhibited B-cell-suppressive activity *in vivo*, similar to that of LAG3^+^ Treg (Supplementary Fig. 3c). We next determined whether the suppression of antibody production via LAG3^+^ Treg is induced not only under lymphopenic conditions, such as Rag1KO mice, but also under more physiological non-lymphopenic conditions. Eα peptide-specific TCR transgenic TEa mice were adoptively transferred with WT B cells and OT-II Th cells and subsequently immunized with NP-OVA once. Co-transferring WT LAG3^+^ Treg effectively suppressed NP-specific antibody production in non-lymphopenic TEa mice (Fig. 1g and Supplementary Fig. 1d). Next, the localization of LAG3^+^ Treg and CD25^+^ Treg was evaluated using Egr2-GFP mice and Foxp3-GFP mice, respectively. As shown in Supplementary Fig. 4a,b, Egr2-GFP^+^CD4^+^ cells were enriched in the T–B-cell border. In contrast, most of Foxp3-GFP^+^CD4^+^ cells were located in the T-cell area. The difference of localization between LAG3^+^ Treg and CD25^+^ Treg may be associated with the functional variation between these regulatory subsets.

### LAG3^+^ Treg suppress a lupus-like disease in MRL*/lpr* mice

**Figure 2:**
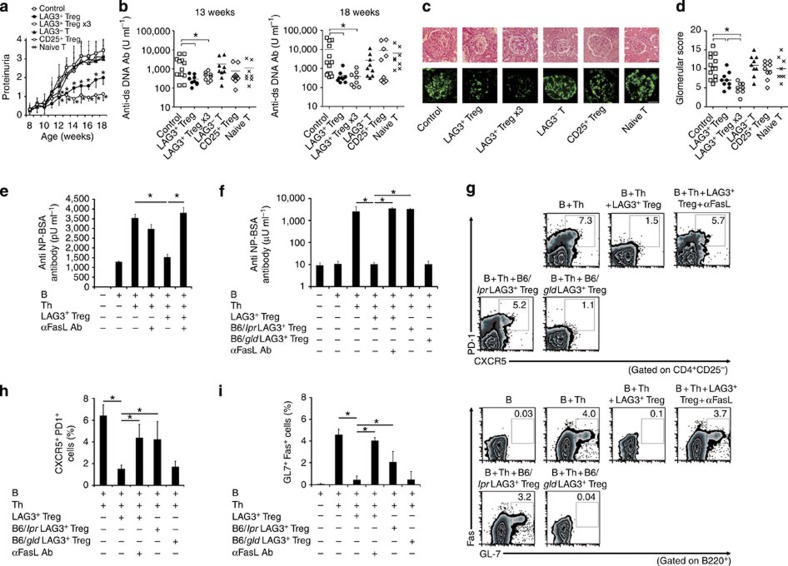
LAG3^+^ Treg regulate B-cell functions through Fas. (**a**–**d**) Treatment of MRL/*lpr* mice with adoptive transfer of T-cell subsets. Ten-week-old MRL/*lpr* mice were injected i.v. with LAG3^+^ Treg (*n*=9), CD4^+^CD25^−^LAG3^−^ T cells (LAG3^−^ T; *n*=9), CD4^+^CD25^+^ Treg (CD25^+^ Treg; *n*=9) or CD4^+^CD25^−^CD45RB^high^ T cells (naive T; *n*=8) from MRL/+ mice (1 × 10^5^ cells each). The mice of LAG3^+^ Treg x3 group (*n*=8) were injected with LAG3^+^ Treg (1 × 10^5^ cells) at 10 weeks of age followed by a twice weekly injection of the same amount of LAG3^+^ Treg. The control group received PBS (*n*=13). (**a**) Proteinuria progression. *P<0.05 versus control group (Mann–Whitney *U*-test). (**b**) Quantification of serum anti-ds DNA antibodies. *P<0.05 (Bonferroni post-test). (**c**) Haematoxylin and eosine (H&E) staining (upper panels) and IgG immunofluorescent staining (lower panels) of kidney sections. Scale bars, 50 μm. (**d**) Glomerular scores. *P<0.05 (Mann–Whitney *U*-test). (**e**) LAG3^+^ Treg-mediated suppression of *in vitro* NP-specific antibody responses. B cells and Th cells purified from NP-OVA/alum-pre-immunized B6 mice and OT-II mice, respectively, were incubated with or without LAG3^+^ Treg from non-immunized OT-II mice in the presence or absence of anti-FasL blocking antibody, and supernatants were analysed for anti-NP-BSA antibodies using ELISA. See also Supplementary Fig. 1e (*n*=6 per group). *P<0.05 (Bonferroni post-test). (**f**) NP-specific antibody responses of Rag1KO mice injected with B6 B cells and OT-II Th cells with or without LAG3^+^ Treg from WT, B6/*lpr* or B6/*gld* mice, as outlined in Fig. 1e (*n*=6 per group). Anti-FasL blocking antibody (200 μg per mouse) was injected i.v. weekly. *P<0.05 (Bonferroni post-test). (**g**–**i**) Flow cytometry plots (**g**) and quantification of splenic CD4^+^CD25^−^CXCR5^+^PD-1^+^ T_FH_ (**h**) and B220^+^GL-7^+^Fas^+^ GCB (**i**) from the same mice as in **f**. Statistical significances in **h**,**i** were analysed by Bonferroni post-test (*P<0.05). The experiments in **e**,**f** were repeated three times. The means±s.d. are indicated.

We investigated whether LAG3^+^ Treg were able to inhibit disease progression in lupus-prone MRL-*Fas*^*lpr/lpr*^ (MRL/*lpr*) mice with a *Fas* mutation^17^. MRL/*lpr* mice were adoptively transferred with one of the various T-cell subsets from Fas-sufficient MRL-*Fas*^+/+^ (MRL/+) mice. LAG3^+^ Treg, but not CD25^+^ Treg, significantly delayed proteinuria progression (Fig. 2a). Furthermore, the three-time transfers of LAG3^+^ Treg almost completely suppressed proteinuria progression. Increases in anti-ds DNA antibody titres and glomerular pathology scores were also inhibited by the single transfer of LAG3^+^ Treg (Fig. 2b–d and Supplementary Fig. 5a). In contrast, consistent with previous reports^18^, the three-time transfers of CD25^+^ Treg from MRL/+ mice to MRL/*lpr* mice did not alter the disease progression (Supplementary Fig. 5b–d). Furthermore, adoptive transfer of LAG3^+^ Treg from MRL/*lpr* mice had no therapeutic benefit in MRL/*lpr* mice (Supplementary Fig. 5b–d). Three-time injections of MRL/+ LAG3^+^ Treg also ameliorated lupus pathologies in MRL/*lpr* mice after the onset of overt proteinuria (Supplementary Fig. 5e,f).

The therapeutic effect of Fas-sufficient MRL/+ LAG3^+^ Treg in Fas-mutated MRL/*lpr* mice suggested that Fas contributes to the suppressive ability of LAG3^+^ Treg. Adding anti-FasL blocking antibody abrogated LAG3^+^ Treg-mediated antibody suppression both *in vitro* (Fig. 2e and Supplementary Fig. 1e) and *in vivo* (Fig. 2f–i). B6/*lpr* LAG3^+^ Treg, but not B6/*gld* LAG3^+^ Treg, failed to suppress antibody production (Fig. 2f–i). Therefore, Fas, but not FasL, on LAG3^+^ Treg is required to suppress B cells. Fas expression on CD4^+^ T cells was independent of Egr2 because activated CD4^+^ T cells from WT and Egr2 CKO mice expressed similar levels of Fas on LAG3^+^ cells (Supplementary Fig. 6).

### TGF-β3 produced by LAG3^+^ Treg controls humoral immunity

**Figure 3:**
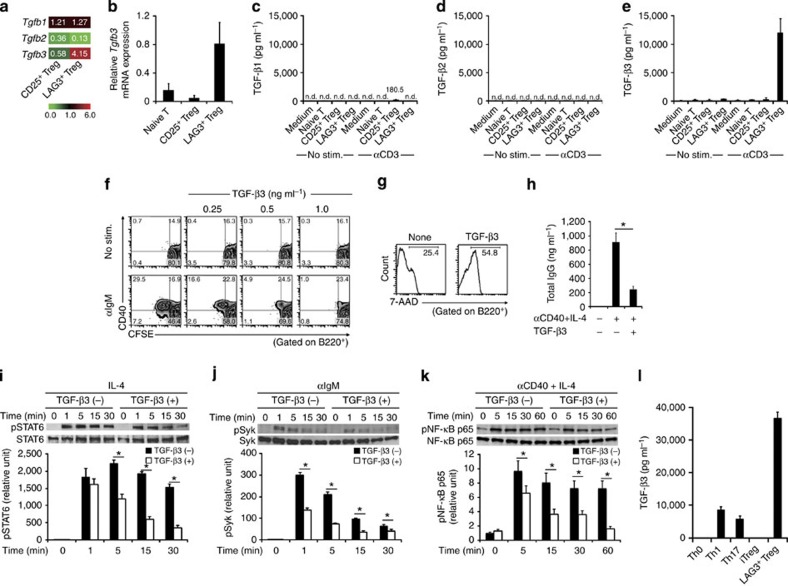
LAG3^+^ Treg suppress B-cell activation through TGF-β3. (**a**) Microarray comparisons of the gene expression profiles between B6 CD25^+^ Treg and B6 LAG3^+^ Treg. Normalized expression values from B6 CD4^+^CD25^−^CD45RB^high^ naive T cells are depicted according to the colour scale shown. (**b**) *Tgfb3* mRNA expression in sorted T-cell subsets taken from the spleens of B6 mice (*n*=3 per group). (**c**–**e**) TGF-β1, 2 and 3 protein levels in the culture supernatants of the indicated T-cell subsets from B6 mice determined using ELISA. Cells were seeded at 1 × 10^5^ cells per well (*n*=4 per group). (**f**) CFSE-labelled B cells were stimulated with or without anti-IgM mAb in the presence or absence of rTGF-β3 (*n*=3 per group). (**g**) Viability of anti-IgM-stimulated B cells in the presence or absence of rTGF-β3 (1 ng ml^−1^) was assessed by 7-AAD (*n*=3 per group). (**h**) The effects of TGF-β3 on total IgG production in the culture supernatants of anti-CD40/IL-4-stimulated B cells, determined as in Fig. 1d (*n*=3 per group). *P<0.05 (unpaired two-tailed Student’s *t*-test). (**i**–**k**) STAT6 (**i**), Syk (**j**) and NF-κB p65 (**k**) phosphorylation in stimulated B cells with or without rTGF-β3, calculated as the ratio of phosphorylated to total protein levels (*n*=3 per group). See also Supplementary Fig. 10. *P<0.05 (unpaired two-tailed Student’s *t*-test). (**l**) TGF-β3 protein levels in the culture supernatants of freshly isolated B6 LAG3^+^ Treg or, naive B6 CD4^+^ T cells cultured under Th0, Th1, Th2 or Th17 conditions determined using ELISA. Cells were seeded at 3 × 10^5^ cells per well (*n*=3 per group). The means±s.d. are indicated.

We next examined whether B-cell suppression by LAG3^+^ Treg is mediated by IL-10 or TGF-β family members. As described above, LAG3 is considered to be one of the specific cell-surface markers for Tr1 cells^16^. We previously reported that LAG3^+^ Treg produce large amounts of IL-10 (ref. 14), and Egr2 mediates IL-27-induced IL-10 production in CD4^+^ T cells through B-lymphocyte-induced maturation protein-1 (Blimp-1; coded by the *Prdm1* gene)^19^. As expected, IL-10 expression levels were significantly reduced in LAG3^+^ Treg from T-cell-specific Prdm1 CKO mice (*Prdm1*^*fl*/*fl*^ CD4-*Cre*^+^) compared with WT mice (Supplementary Fig. 7a). Although LAG3^+^ Treg derived from Prdm1 CKO and IL-10-deficient (IL-10KO) mice showed a slight reduction in the suppressive activity for *in vivo* NP-specific antibody responses compared with WT LAG3^+^ Treg, there were no statistical differences in suppressive activity among these three LAG3^+^ Treg (Supplementary Fig. 7b). These results suggested that IL-10 is not critical for B-cell suppression by LAG3^+^ Treg. Microarray analysis^14^ and quantitative real-time PCR of LAG3^+^ Treg revealed a significant increase in TGF-β3 expression, but not TGF-β1 or 2 (Fig. 3a,b). TCR stimulation induced the production of a large amount of TGF-β3, but not TGF-β1 or 2, in the culture supernatants of LAG3^+^ Treg (Fig. 3c–e). The same trend was observed for the differences in mRNA levels of *Tgfb* families in LAG3^+^ Treg (Supplementary Fig. 8). In contrast, CD25^+^ Treg only produced small amounts of TGF-β1 under these conditions. TGF-β3 markedly suppressed anti-IgM-stimulated B-cell proliferation and CD40 expression (Fig. 3f), strongly induced B-cell death (Fig. 3g), and suppressed total IgG production (Fig. 3h). TGF-β3 produced similar effects as TGF-β1 and 2 (Supplementary Fig. 9a,b), in accordance with previous findings that TGF-β1 strongly suppresses B-cell functions^20^. Regarding signal transduction, the addition of TGF-β3 significantly reduced the phosphorylation of signal transducer and activator of transcription (STAT) 6, Syk and NF-κB p65 in activated B cells (Fig. 3i–k and Supplementary Fig. 10). IL-4 produced by Th cells enhances the proliferation and survival of B cells while promoting immunoglobulin secretion and isotype switching via the activation of STAT6 (ref. 21). Activation of the tyrosine kinase Syk is critical for the cell signalling in response to B-cell receptor stimulation^22^. Activation of CD40, which is required for specific antibody production by antigen-stimulated B cells, induces phosphorylation of NF-κB p65 (ref. 23). Therefore, TGF-β3 inhibits several important pathways for B-cell functions. TGF-β3 production is not limited to LAG3^+^ Treg because TGF-β3 is also produced by developing Th17 cells in an IL-23-dependent manner^24^. We found that Th1 cells produced TGF-β3 in addition to Th17 cells (Fig. 3l). However, LAG3^+^ Treg produced significantly greater amounts of TGF-β3 than Th1 and Th17 cells.

**Figure 4:**
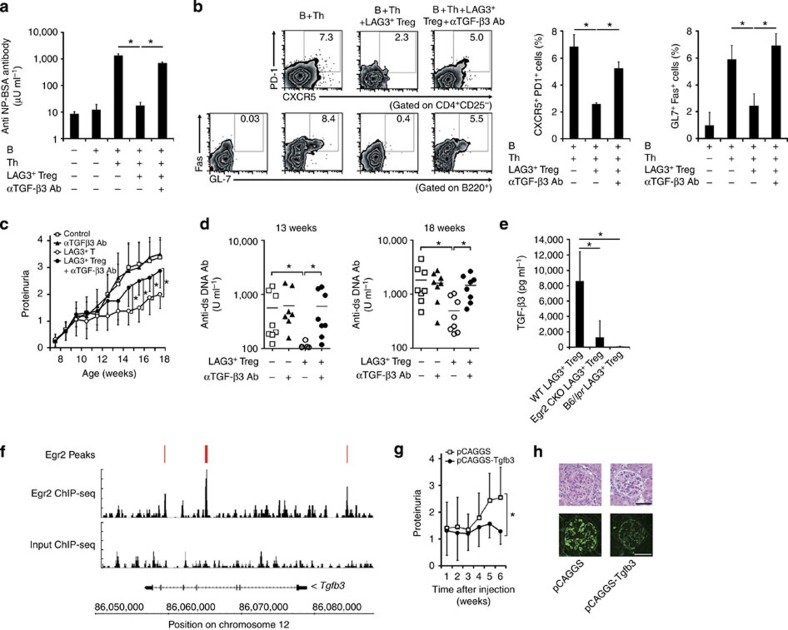
TGF-β3 ameliorates lupus manifestations. (**a**) *In vivo* blockade of LAG3^+^ Treg-mediated B-cell suppression by weekly injections of anti-TGF-β3 blocking mAb (100 μg per mouse) in NP-OVA-immunized Rag1KO mice transferred with B cells and T cells, as outlined in Fig. 1e (*n*=6 per group). *P<0.05 (Bonferroni post-test). (**b**) Flow cytometry plots and quantification of splenic CD4^+^CD25^−^CXCR5^+^PD-1^+^ T_FH_ and B220^+^GL-7^+^Fas^+^ GCB from the same mice as in **a**. *P<0.05 (Bonferroni post-test). (**c**,**d**) Proteinuria progression (**c**) and serum levels of anti-dsDNA antibody (**d**) in MRL/+ LAG3^+^ Treg-transferred MRL/*lpr* mice with or without a weekly injection of anti-TGF-β3 mAb (100 μg per mouse; *n*=8 mice per group). Statistical significances in **c** were analysed with Mann–Whitney *U*-test, and **d** were analysed by Bonferroni post-test (*P<0.05). (**e**) Production of TGF-β3 by anti-CD3-stimulated LAG3^+^ Treg from WT, Egr2 CKO or B6/*lpr* mice, as in Fig. 3e (*n*=6 per group). *P<0.05 (Bonferroni post-test). (**f**) Distribution of Egr2-binding sites revealed by ChIP-seq in LAG3^+^ Treg. Egr2 ChIP-seq signal and Input ChIP-seq signal tracks are shown together with Egr2 peak calls (Egr2 Peaks). Peaks were identified using the Bioconductor package BayesPeak. (**g**) Proteinuria progression in MRL/*lpr* mice after i.v. injection with pCAGGS control (*n*=8) or pCAGGS-Tgfb3 plasmid vector (*n*=7). *P<0.05 (Mann–Whitney *U*-test). (**h**) Representative images of kidney sections subjected to H&E staining (upper panels) and IgG immunofluorescent staining (lower panels) from the same mice as in Fig. 2c. Scale bars, 50 μm. The experiments in **e** were repeated three times.

Treatment with a TGF-β3 blocking antibody cancelled the LAG3^+^ Treg-mediated suppression of antibody production and the development of T_FH_ and GCB in Rag1KO mice transferred with WT B cells and WT OT-II Th cells (Fig. 4a,b). Although TGF-β1-LAP was detected on stimulated LAG3^+^ Treg (Supplementary Fig. 11a), TGF-β1 blockade did not affect the suppressive activity of LAG3^+^ Treg for T_FH_ and GCB development and antibody production (Supplementary Fig. 11b,c). There was a possibility that reduction of antigen-specific IgG class antibody by LAG3^+^ Treg was related to the class switching to IgA because TGF-β and TGF-β receptor II (TGFβRII) signalling induce IgA class switching^25^,^26^,^27^. However, no induction of antigen-specific IgA class antibody was observed by the co-transfer of WT LAG3^+^ Treg to Rag1KO mice transferred with WT B cells and WT OT-II Th cells (Supplementary Fig. 11d). TGF-β3 blockade also abrogated the therapeutic effects of MRL/+ LAG3^+^ Treg in MRL/*lpr* mice (Fig. 4c,d), indicating a critical role for TGF-β3. Intriguingly, TGF-β3 production by Egr2-deficient LAG3^+^ Treg and Fas-mutated B6/*lpr* LAG3^+^ Treg was markedly reduced (Fig. 4e). Therefore, Egr2 and Fas are required for the production of TGF-β3 and the B-cell-suppressive activity of LAG3^+^ Treg. Intriguingly, Fas-deficient B6/*lpr* mice contained LAG3^+^ Treg that express *Tgfb3* mRNA (Supplementary Fig. 12a,b), which indicated that Fas expression is not necessary for *Tgfb3* mRNA transcription in LAG3^+^ Treg. To further investigate the dependence of Egr2 on the transcription of Tgfb3, we performed Egr2 chromatin immunoprecipitation (ChIP)-seq analysis in LAG3^+^ Treg. The ChIP-seq analysis identified the binding sites of Egr2 in *Tgfb3* loci, suggesting direct regulation of the *Tgfb3* gene by Egr2 (Fig. 4f). The importance of TGF-β3 for the control of lupus in MRL/*lpr* mice has also been verified by the observation that gene delivery of a TGF-β3-expressing plasmid significantly improved proteinuria progression and renal pathology (Fig. 4g,h).

### LAG3^+^ Treg-mediated B-cell suppression requires PD-1

**Figure 5:**
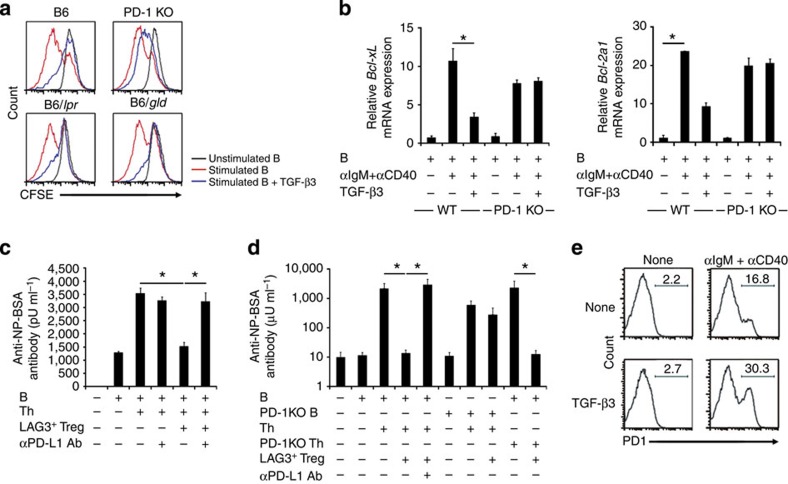
PD-1 expression on B cells is important for the suppressive activity of LAG3^+^ Treg. (**a**) Resistance to TGF-β3-mediated B-cell suppression in PD-1-deficient (PD-1KO) mice. CFSE-labelled B cells from B6, PD-1KO, B6/*lpr* or B6/*gld* mice were stimulated *in vitro* for 72 h with anti-IgM and anti-CD40 in the presence or absence of rTGF-β3 (1 ng ml^−1^). Histograms are gated on B220^+^ B cells. (**b**) *Bcl-xL* and *Bcl-2a1* mRNA levels in anti-IgM-stimulated B cells from WT or PD-1KO mice in the presence or absence of rTGF-β3 (1 ng ml^−1^; *n*=3 per group). *P<0.05 (unpaired two-tailed Student’s *t*-test). (**c**) Blockade of LAG3^+^ Treg-mediated suppression of *in vitro* NP-specific antibody responses by anti-PD-L1 blocking mAb. B cells and Th cells purified from NP-OVA/alum-pre-immunized B6 mice and OT-II mice, respectively, were incubated with or without LAG3^+^ Treg from non-immunized OT-II mice in the presence or absence of anti-PD-L1 blocking mAb, and supernatants were analysed for anti-NP-BSA antibodies by ELISA (*n*=6 per group). *P<0.05 (Bonferroni post-test). (**d**) NP-specific antibody responses of Rag1KO mice injected with B6 B cells and OT-II Th cells from B6 or PD-1KO mice with or without LAG3^+^ Treg from B6 mice. Anti-PD-L1 blocking antibody (200 μg per mouse) was injected i.v. every 3 days. Anti-NP-BSA antibody levels were determined as in Fig. 1e (*n*=6 per group). *P<0.05 (Bonferroni post-test). (**e**) PD-1 expression on B cells. B cells from B6 mice were stimulated *in vitro* for 72 h with or without anti-IgM and anti-CD40 antibodies in the presence or absence of rTGF-β3 (1 ng ml^−1^). Histograms are gated on B220^+^ B cells. Data are representative of three independent experiments. The means±s.d. are indicated.

Genetic variants of the programmed cell death-1 (*PD-1*) gene have been associated with SLE susceptibility^1^. PD-1 provides negative co-stimulatory signals to both T cells and B cells^28^,^29^, and PD-1-deficient (PD-1KO) mice develop a lupus-like disease^30^. Furthermore, PD-1 and LAG3 synergistically regulate autoimmunity and tumour immunity^31^,^32^. To examine the potential cooperation between TGF-β3 and PD-1, we added TGF-β3 to anti-IgM-stimulated B cells from B6, PD-1KO, Fas-deficient B6/*lpr* and FasL-deficient B6/*gld* mice. PD-1KO B cells, but not B6/*lpr* or B6/*gld* B cells, showed a partial resistance to the TGF-β3-induced inhibition of cell division (Fig. 5a). Expressions of TGF-βRII, a receptor for TGF-β3, on both T cells and B cells were not different among B6, PD-1 KO, B6/*lpr* and B6/*gld* mice (Supplementary Fig. 13). In accordance with a previous report that phosphorylated STAT6 is known to induce the expression of anti-apoptotic Bcl-xL in B-cell lines^33^, TGF-β3 suppressed the expression of Bcl-xL and Bcl-2a1 in activated WT B cells, but not in PD-1KO B cells (Fig. 5b). The cooperative suppression of B cells by TGF-β3 and PD-1 underscores the importance of PD-1 expression on B cells for LAG3^+^ Treg-mediated suppression. The addition of an anti-PD-L1 blocking antibody reversed the LAG3^+^ Treg-mediated suppression of antibody production *in vitro* (Fig. 5c). Anti-NP antibody production was not suppressed by the co-transfer of WT LAG3^+^ Treg in Rag1KO mice transferred with PD-1KO B and WT OT-II Th cells; however, Rag1KO mice transferred with WT B cells and PD-1KO OT-II Th cells were susceptible to LAG3^+^ Treg-mediated suppression (Fig. 5d). Notably, TGF-β3 significantly enhanced the expression of PD-1 on B cells stimulated with anti-IgM and anti-CD40 antibody (Fig. 5e). The interaction of PD-1 and its ligand, PD-L1, may participate in the amplification of signalling cascade driven by TGF-β3, which is essential for LAG3^+^ Treg-mediated B-cell suppression. These results confirm that PD-1 expression on B cells is required for LAG3^+^ Treg-mediated B-cell suppression. CD4^+^CD25^−^Egr2^+^T cells co-expressed both PD-L1, the ligand for PD-1, and LAG3 (Supplementary Fig. 14a,b). However, because PD-L1 and PD-L2 are expressed on various cell types including GCB^34^, PD-L1 on LAG3^+^ Treg may not be the only source of ligands for PD-1 on B cells.

### IL-27 induces TGF-β3-producing Egr2^+^ T cells

**Figure 6:**
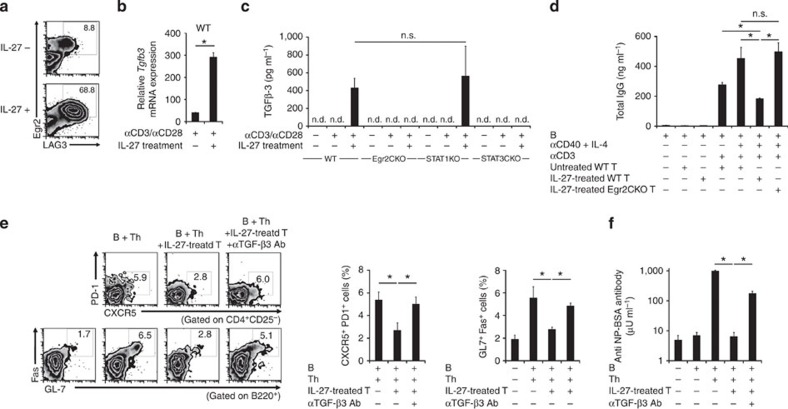
IL-27 induces TGF-β3-producing Egr2^+^ Treg from naive T cells. (**a**) IL-27-mediated induction of Egr2 and LAG-3 on CD4^+^ T cells. Freshly isolated naive CD4^+^ T cells were stimulated with anti-CD3/CD28 mAb in the presence or absence of IL-27. Cells were stained for Egr2 and LAG-3 expression on day 5. (**b**) Quantitative RT–PCR analysis of *Tgfb3* mRNA expression in naive WT CD4^+^ T cells activated as in **a**, assessed on day 3. *P<0.05 (unpaired two-tailed Student’s *t*-test). (**c**) ELISA for TGF-β3 in culture supernatants of activated naive WT, Egr2 CKO, STAT1 KO, or *Stat3*^*fl*/*fl*^ CD4-*Cre*^+^ (STAT3 CKO) CD4^+^ T cells as in **a**, assessed on day 5. *P<0.05 (Bonferroni post-test). (**d**) *In vitro* B-cell suppression by IL-27-treated naive WT CD4^+^ T cells. IL-27-treated or untreated naive WT or Egr2 CKO CD4^+^ T cells stimulated with anti-CD3 mAb were co-cultured with anti-CD40/IL-4-stimulated B cells. Total IgG was determined in culture supernatants on day 7 by ELISA (*n*=3 per group). *P<0.05 (Bonferroni post-test). (**e**) Flow cytometry plots and quantification of splenic CD4^+^CD25^−^CXCR5^+^PD-1^+^ T_FH_ and B220^+^GL7^+^Fas^+^ GCB. B6 B cells and OT-II Th cells were transferred with or without IL-27-treated T cells into Rag1KO mice immunized twice with NP-OVA/alum, as in Fig. 1e (*n*=6 per group). Anti-TGF-β3 blocking antibody (100 μg per mouse) was injected i.v. weekly. Numbers indicate the percentage of cells contained within the rectangular regions. *P<0.05 (Bonferroni post-test). (**f**) Serum levels of anti-NP-specific IgG1 antibody from the same mice as in **e**. *P<0.05 (Bonferroni post-test). n.d., not detected; n.s., not significant. Data are representative of three independent experiments. The means±s.d. are indicated.

IL-27 is a member of the IL-12/IL-23 heterodimeric family of cytokines produced by antigen-presenting cells (APCs). IL-27 has been identified as a differentiation factor for IL-10-producing Tr1 cells^35^. We have previously reported that IL-27 induces Egr2 expression in CD4^+^ T cells, and Egr2 is required for the Blimp-1-mediated IL-10 production^19^. IL-27 treatment induced not only Egr2 but also a significant amount of TGF-β3 protein (Fig. 6a,b). Egr2-deficient CD4^+^ T cells exhibited a substantial reduction in IL-27-induced production of TGF-β3 (Fig. 6c), confirming the importance of Egr2 for the induction of TGF-β3. The activation of specific STAT proteins in CD4^+^ T cells is associated with the differentiation of helper T-cell lineages. Although IL-27-mediated IL-10 induction requires both STAT1 and STAT3 (ref. 35), we previously found that IL-27-mediated induction of Egr2 is dependent on STAT3 (ref. 19). IL-27-mediated TGF-β3 induction was impaired in STAT3-, not STAT1-, deficient CD4^+^ T cells (Fig. 6c), demonstrating similarity between Egr2 and TGF-β3 in STAT3 dependency. Moreover, IL-27-treated CD4^+^ T cells significantly suppressed B-cell antibody production (Fig. 6d). In contrast, Egr2-deficient CD4^+^ T cells treated with IL-27 failed to suppress antibody production by B cells. IL-27-treated CD4^+^ T cells also exhibit suppressive activity for T_FH_ and GCB development and antibody production in a TGF-β3-dependent manner (Fig. 6e,f). These results suggested that IL-27 induces CD4^+^ T cells that share several characteristics with LAG3^+^ Treg. Thus, IL-27-induced TGF-β3-producing cells exhibit suppressive activity on humoral immunity in an Egr2-dependent manner.

### Human LAG3^+^ Treg suppress antibody production

**Figure 7:**
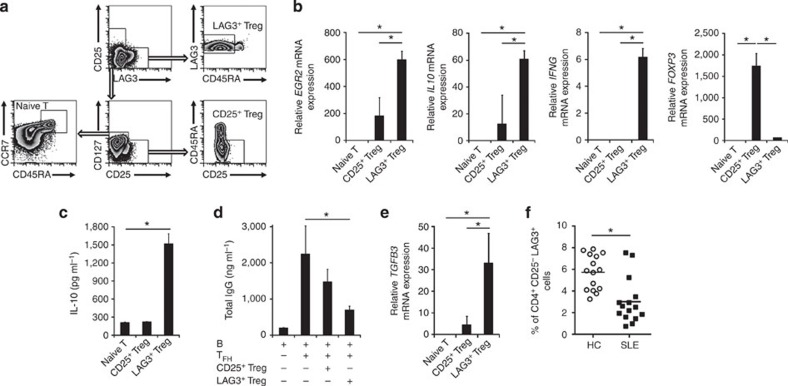
Human CD4^+^CD25^−^CD45RA^−^LAG3^+^ T cells suppress antibody production. (**a**) Gating strategy for CD4^+^CD25^−^CD127^high^CCR7^+^ T cells (naive T), CD4^+^CD25^high^CD127^low^CD45RA^−^ T cells (CD25^+^ Treg) and CD4^+^CD25^−^CD45RA^−^LAG3^+^ T cells (LAG3^+^ Treg). Freshly isolated human PBMCs from healthy controls (HCs) were stained for CD4, CD25, CD45RA, CD127, CCR7 and LAG3, and the percentages of cells in each quadrant are indicated. (**b**) Quantitative RT–PCR analysis of *EGR2*, *IL10*, *IFNG* and *FOXP3* mRNA expression in anti-CD3 mAb-stimulated conditions for each CD4^+^ T-cell subset from HC (*n*=3 per group). *P<0.05 (Bonferroni post-test). (**c**) IL-10 protein levels in the culture supernatants on day 7 of the indicated T-cell subsets determined by ELISA (*n*=3 per group). *P<0.05 (unpaired two-tailed Student’s *t*-test). (**d**) *In vitro* B-cell suppression by each CD4^+^ T-cell subset from HC. Each CD4^+^ T-cell subset was co-cultured with T_FH_ and staphylococcal enterotoxin B (SEB)-stimulated B cells. Total IgG was determined in culture supernatants by ELISA (*n*=3 per group). *P<0.05 (Bonferroni post-test). (**e**) *TGFB3* mRNA expression in sorted T-cell subsets taken from HC (*n*=3). *P<0.05 (Bonferroni post-test). (**f**) Percentages of CD4^+^CD25^−^CD45RA^−^LAG3^+^ T cells in each HC (*n*=15) and SLE patients (*n*=15) as in **a**. *P<0.05 (unpaired two-tailed Student’s *t*-test). The means±s.d. are indicated.

We identified CD4^+^CD25^−^CD45RA^−^LAG3^+^ T cells in CD4^+^ T cells from peripheral blood mononuclear cells (PBMCs) of healthy donors (Fig. 7a). Similar to murine LAG3^+^ Treg, human CD4^+^CD25^−^CD45RA^−^LAG3^+^ T cells expressed *EGR2*, *IL10* and *IFNG* (Fig. 7b) and produced significant amounts of IL-10 in response to TCR stimulation (Fig. 7c). The regulatory activity of human CD4^+^CD25^−^CD45RA^−^LAG3^+^ T cells was confirmed by the observation that they more efficiently suppressed antibody production when co-cultured with B cells and T_FH_ cells compared with CD4^+^CD25^+^CD127^low^CD45RA^−^-activated Treg^36^ (Fig. 7d). Human CD4^+^CD25^−^CD45RA^−^LAG3^+^ T cells expressed high levels of TGF-β3 (Fig. 7e), suggesting that they suppress B cells through a mechanism identical to murine LAG3^+^ Treg. Therefore, we consider the CD4^+^CD25^−^CD45RA^−^LAG3^+^ T-cell population as the human counterpart to murine LAG3^+^ Treg. We next assessed whether the frequency of LAG3^+^ Treg might be reduced in human systemic autoimmunity. The percentages of circulating LAG3^+^ Treg were significantly lower in the peripheral blood of SLE patients compared with healthy donors (Fig. 7f). These findings suggest that LAG3 expression could be used for tracking of the T-cell population with antibody-suppressing capacity in SLE patients.

## Discussion

The results of the present study demonstrated that LAG3^+^ Treg suppress the development of GCB and T_FH_, antibody production and disease progression in lupus-prone MRL/*lpr* mice. TGF-β3, which is produced by LAG3^+^ Treg in Egr2- and Fas-dependent manners, plays a critical role in suppressing humoral immunity. As LAG3^+^ Treg also produce much higher levels of IL-10 than CD25^+^ Treg^14^, LAG3^+^ Treg are potent producers of regulatory cytokines. The pro-inflammatory role of TGF-β3 was previously demonstrated by the observation that TGF-β3 efficiently induces pathogenic Th17 cells^24^,^37^. Our results have revealed a previously unrecognized role for TGF-β3 in the control of autoimmunity. TGF-β1 also exerts both pro-inflammatory and anti-inflammatory effects^27^,^38^,^39^. In particular, TGF-β1 induces B-cell apoptosis and reduces immunoglobulin production from activated human tonsil B cells^40^,^41^. CD25^+^ Treg and Th3 regulatory cells^42^,^43^ are potent sources of TGF-β1, and CD25^+^ Treg have been shown to suppress B-cell immunoglobulin synthesis through TGF-β1 (ref. 44). However, the amount of TGF-β1 produced by CD4^+^ T cells including CD25^+^ Treg is relatively limited (Fig. 3c), and it has been difficult to define the sources of TGF-β1 that are relevant to immune suppression. Although it was demonstrated in a number of systems that TGF-β1 and TGF-β3 display clear isoform-specific biology, TGF-β1 and TGF-β3 showed comparable suppressive activity on B-cell responses (Supplementary Fig. 9a,b). In terms of helper T-cell development, TGF-β3 is autonomously produced by Th17 cells during the development of pathogenic Th17 cells^24^. In our setting, not only Th17 cells but also Th1 cells produced significant amounts of TGF-β3; however, LAG3^+^ Treg produced greater amounts of TGF-β3 compared with Th1 and Th17 cells (Fig. 3l). Therefore, the large amount of TGF-β3 produced by LAG3^+^ Treg plays a significant role in the generation and maintenance of immune tolerance. As IL-10 strongly suppresses Th17 development and function^45^, IL-10 produced by LAG3^+^ Treg may counteract the pro-inflammatory aspect of TGF-β3.

We identified two molecules, Fas and Egr2, that are required for TGF-β3 secretion in LAG3^+^ Treg. Egr2 deficiency in T cells and B cells results in a lupus-like syndrome, and Egr2 directly activates p21^cip1^ expression in CD44^high^ T cells and is involved in the control of Th1 and Th17 differentiation^11^. The fact that Egr2 blocks the function of BATF, an AP-1 inhibitor required for the differentiation of Th17 cells, indicates that Egr2 is an intrinsic regulator of effector T cells^12^. We showed here that Egr2 in T cells is important for the control of the development of T_FH_ and GCB (Fig. 1). Extrinsic functions of Egr2 for regulating humoral immunity were confirmed by our observation that transfer of Egr2-sufficient LAG3^+^ Treg suppressed the excessive expansion of T_FH_ and GCB as well as antibody production in Egr2 CKO mice (Fig. 1). Fas controls T- and B-cell expansion by triggering apoptosis. The *lpr* gene, a mutation in the *Fas* gene in which the insertion of an early transposon results in a splicing error, interferes with peripheral T-cell tolerance, prevents Fas-dependent elimination of anergic B cells by CD4^+^ T cells and facilitates provision of T-cell help to autoreactive B cells. The impaired production of TGF-β3 by LAG3^+^ Treg may contribute to autoimmunity in MRL*/lpr* mice, as exogenous supplementation of TGF-β3 ameliorated the disease (Fig. 4g,h). These findings may indicate that Fas exhibits its tolerogenic activity via more complex regulatory pathways than previously thought.

IL-27, a differentiation factor for IL-10-producing Tr1 cells^35^, induces CD4^+^Egr2^+^LAG3^+^ T cells^19^. However, the role of Tr1 cells in the regulation of B cells is not clear because CD46-induced Tr1 cells are more potent at enhancing immunoglobulin production compared with conventional T cells^46^. In LAG3^+^Treg, IL-10 did not directly contribute to the control of B-cell response (Supplementary Fig. 7). Nevertheless, the linkage between IL-27 and the control of antibody production was suggested by the observation that overexpression of the IL-27 receptor, WSX-1, protects MRL/*lpr* mice from the development of autoimmune disease^47^. It is notable that, whereas STAT1 and STAT3 are required for the induction of IL-10 by IL-27, the ability of IL-27 to promote Egr2 and TGF-β3 is STAT3-dependent (Fig. 6c). As STAT3-acitvating IL-6 also induces TGF-β3 production^24^, STAT3 may play key roles for TGF-β3 induction in CD4^+^ T cells.

We here described potential cooperation of four molecules, Egr2, Fas, TGF-β3 and PD-1, in the control of humoral immunity. Activities of TGF-β family molecules are regulated by not only transcription but also protein-processing with proteases and integrins^37^. Although the precise relationships remain to be clarified, Egr2 and Fas may regulate transcription and protein-processing of TGF-β3. The recruitment of SHP-2 phosphatase to the phosphorylated Tyr residue in PD-1 is in part responsible for the inhibitory effect through the dephosphorylation of signalling molecules belonging to the TCR or B-cell receptor pathways^48^. Likewise, PD-1 signalling may modulate intracellular cascade downstream of TGF-β3.

Mouse and human CD25^+^ Treg share most of their features, although several differences exist regarding subset specificities and marker molecules. Human LAG3^+^ Treg express many key molecules in common with mouse LAG3^+^ Treg, and both suppress antibody production *in vitro*. Despite considerable efforts to clarify the contribution of CD25^+^ Treg to the development of SLE in patients, their role in generating disease remains elusive. However, Miyara *et al*.^36^ reported that the number of CD4^+^CD25^+^CD127^low^CD45RA^−^-activated Treg is decreased with a notable concomitant increase in the Foxp3^low^CD45RA^−^ memory/effector-like non-Treg subset in active SLE. Several reports have described quantitative and qualitative reduction of CD25^+^ Treg in SLE^49^. The functional impairment of immune-regulatory mechanisms may be crucial for the initiation and perpetuation of autoimmune disease, and a reduced frequency of LAG3^+^ Treg in SLE could also play a role in the dysregulated autoantibody production. Collectively, LAG3^+^ Treg may also be a regulatory mechanism for pathogenic autoantibody production in addition to CD25^+^ Treg. A deeper understanding of LAG3^+^ Treg will be useful for the treatment of autoantibody-mediated autoimmune diseases including SLE.

## Methods

### Mice

C57BL/6 (B6), C57BL/6-Fas^*lpr*/*lpr*^ (B6/*lpr*), C57BL/6-FasL^*gld*/*gld*^ (B6/*gld*), MRL-Fas^*lpr/lpr*^ (MRL/*lpr*) and MRL-Fas^+/+^ (MRL/+) mice were purchased from Japan SLC. B6 recombinase-activating gene (*Rag*)-1-deficient (Rag1KO) mice, floxed-*Prdm1* (*Prdm1*^*fl/fl*^) mice, *Il-10*-deficient (IL-10KO) mice, TCR transgenic OT-II mice (specific for the chicken ovalbumin peptide (amino-acid residues 323–339) in the context of MHC class II I-A^b^) and TEa mice (specific for the Eα peptide (amino-acid residues 52–68) from the MHC class II I-Eα molecule in the context of I-A^b^) were purchased from Jackson Laboratories. Floxed-*Stat3* (*Stat3*^*fl/fl*^) mice were purchased from Oriental Bio Service (Japan). Rag1KO mice were housed in microisolator cages with sterile filtered air. B6-*Pdcd1*-deficient (PD-1KO) mice^47^ were purchased from RIKEN BRC (Japan). Floxed *Egr2* (*Egr2*^*fl/fl*^) mice were provided by Patrick Charnay (INSERM, France)^48^. *Egr2* CKO mice (*Egr2*^*fl*/*fl*^ CD4-*Cre*^+^), Prdm1 CKO mice (*Prdm1*^*fl*/*fl*^ CD4-*Cre*^+^) and STAT3 CKO (*Stat3*^*fl*/*fl*^ CD4-*Cre*^+^) mice were generated by crossing *Egr2*^*fl/fl*^ mice, *Prdm1*^*fl*/*fl*^ mice or *Stat3*^*fl/fl*^ mice with CD4-*Cre* transgenic mice on a B6 background, respectively. CD4-*Cre* transgenic mice (line 4196), originally generated by C. B. Wilson and colleagues, and *Stat1*-deficient (STAT1KO) mice were purchased from Taconic. Age- and sex-matched mice that were ≥7 weeks of age were used for all experiments. All animal experiments were approved by the ethics committee of the University of Tokyo Institutional Animal Care and Use Committee.

### Reagents, antibodies and medium

The following reagents were purchased from BD Pharmingen; purified monoclonal antibody (mAb) for CD3ε (145-2C11), FasL blocking (MFL3), anti-CD40 (3/23), Fc block (anti-CD16/CD32, 1.5:100 dilution), fluorescein isothiocyanate (FITC) anti-CD45RB (16A, 2:100), FITC anti-Fas (Jo2, 2:100), phycoerythrin (PE) anti-CD45RB (16A, 1:100), APC-Cy7 anti-CD45RB (16A, 2:100), PE anti-LAG3 (C9B7W, 3:100), APC anti-LAG3 (C9B7W, 3:100), FITC anti-IgG1 (A85-1, 2:100), APC anti-IgG1 (A85-1, 2:100), FITC anti-GL7 (Ly-77, 0.5:100), FITC anti-CD25 (PC61, 2:100), PE anti-CD25 (PC61, 1:100), APC anti-CD25 (PC61, 1:100), APC-Cy7 anti-CD25 (PC61, 1:100), APC anti-CD4 (L3T4, 1:100), APC-Cy7 anti-CD4 (L3T4, 1:100), PE anti-CXCR5 (2G8, 5:100), APC-Cy7 anti-B220 (RA3-6B2, 1:100), PE anti-CD40 (3/23, 1:100), PE anti-PD-1 (J43, 1:100), biotinylated mAb for CD8a (53-6.7), CD19 (1D3), CD11c (HL3), CD45RB (16A), CD25 (7D4) and CXCR5 (2G8), streptavidin (SA)-FITC antibody (Ab), SA-APC and SA-APC-Cy7. Alexa Fluor 488 anti-LAG3 mAb (C9B7W, 1:100) and FITC anti-PD-L1 mAb (MIH6, 2:100) were purchased from AbD Serotec. Qdot605 anti-CD4 mAb (RM4-5, 2:100) and SA-Qdot605 were purchased from Invitrogen. PE anti-PD-L1 mAb (MIH5, 1:100), PE anti-Egr2 mAb (erongr2, 1:100), PE anti-PD-L1 mAb (10F. 9G2, 1:100) and APC anti-B220 mAb (RA3-6B2, 1:100) were purchased from eBioscience. NP(13)-OVA and NP(9)-BSA were purchased from Biosearch Technologies. SA-conjugated microbeads were purchased from Miltenyi Biotec. FITC anti-mouse IgG Ab was purchased from Sigma. BrilliantViolet421 anti-B220 mAb, APC anti-mouse LAP (Tgf-β1, 2:100) and anti-PD-L1 blocking mAb (10F.9G2) were purchased from Biolegend. Alexa 488 Fluor anti-GFP mAb was purchased from Medical & Biological Laboratories. Recombinant TGF-β1 (rTGF-β1) and three were purchased from Miltenyi Biotec, and rTGF-β2, anti-TGF-β1 blocking polyclonal Ab (MAB240), anti-TGF-β3 blocking polyclonal Ab (MAB234), APC anti-mouse TGF-βRII (1.5:100), APC goat IgG and rIL-27 were purchased from R&D Systems.

For human studies the following anti-human mAbs were used: V450 anti-hCD4 (RPA-T4, 2:100), V500 anti-hCD4 (RPA-T4, 2:100; both from BD Biosciences), PerCP-Cy5.5 anti-human CD3 (UCHT1, 2:100), Brilliant Violet 421 anti-hCD25 (BC96, 3:100), APC anti-hCD19 (HIB19, 2:100), PerCP-Cy5.5 anti-hCD4 (OKT4, 5:100), Alexa Fluor 647 anti-hCD197 (G043H7, 3:100), APC-Cy7 anti-human CD45RA (HI100, 3:100; all from BioLegend), Alexa Fluor 488 anti-hCD25 (BC96, 4:100) and PE-Cy7 anti-hCD127 (eBioRDR5, 1:100; all from eBioscience). PE anti-hLAG3 polyclonal Ab (10:100) was purchased from R&D Systems.

T cells and B cells were cultured in RPMI-1640 medium supplemented with 10% FBS, 100 μg ml^−1^ L-glutamine, 100 U ml^−1^ penicillin, 100 μg ml^−1^ streptomycin and 50 μM 2-mercaptoethanol (all purchased from Sigma).

### Generation of Egr2-GFP mice

The bacterial artificial chromosome (BAC) clone RP23-88D4, which contained the entire genomic *Egr2* locus, was obtained from BAC Libraries (Invitrogen). This clone was modified for the insertion of an enhanced GFP gene (eGFP) with an SV40 polyA sequence at the initiation codon in Egr2 exon 1 using the Red/ET recombination system. The Egr2-eGFP construct linearized with PI-SceI was injected into the pronuclei of fertilized zygotes from B6 mice and transferred to pseudopregnant females. Offsprings were screened for genomic integration by PCR of tail DNA using the following Egr2 promoter primer: forward 5′- AGACCGCATTTACTCTTATCACCAG -3′, SV40polyA-specific primer: reverse 5′- TGAGTTTGGACAAACCACAACTAGA -3′ (PCR product size: 2.1 kb). Mice were generated by breeding F1 heterozygous transgenic males with WT females.

### Cell purification

Briefly, spleens were cut into pieces and digested with collagenase type IV (Sigma-Aldrich). Red blood cells were lysed with hypotonic shock induced by brief exposure to ammonium chloride with potassium lysis buffer, followed by immediate isotonic restoration. Surface staining was performed in ice-cold PBS with 2% fetal calf serum in the presence of an FcR-blocking antibody (anti-mouse CD16/CD32 mAb). To obtain highly purified CD4^+^ T cells, single-cell suspensions were first purified by negative selection with magnetic-activated cell sorting (MACS; Miltenyi Biotec) using anti-B220 mAb, anti-CD19 mAb, anti-CD11c mAb and anti-CD8a mAb. To obtain highly purified CD4^+^CD25^−^CD45RB^low^LAG3^+^ T cells, CD45RB^high^ cells were subsequently depleted with anti-CD45RB mAb. After FcR-blocking, the prepared cells were stained with mAbs specific for CD4, CD25, CD45RB and LAG3 in order to isolate CD4^+^CD25^−^CD45RB^low^LAG3^+^ T cells (LAG3^+^ Treg), CD4^+^CD25^+^ T cells (CD25^+^ Treg), CD4^+^CD25^−^LAG3^−^ Th cells or CD4^+^CD25^−^CD62L^hi^CD44^low^ (naive T). Cells for intracellular anti-Egr2 staining were stained using the Foxp3 Staining Buffer Set (eBioscience) according to the manufacturer’s protocol. The purities of MACS- and FACS (FACSVantage SE (Becton-Dickinson) or MoFlo XDP (Beckman Coulter))-sorted cells were >90% and >99%, respectively.

### Immunization

NP-OVA in alum was prepared by mixing NP(13)-OVA (Biosearch Technologies) in PBS with alum (Pierce) at a 1:1 ratio for 30 min at 4 °C. The immunization with NP-OVA/alum was performed by intraperitoneal injection.

### Adoptive transfer of LAG3^+^ Treg from WT mice into Egr2 CKO mice

FACS-purified 2 × 10^5^ LAG3^+^ Treg from B6 mice were injected intravenous (i.v.) into 10-week-old Egr2 CKO mice pre-immunized with or without 100 μg NP-OVA/alum 1 day before the cell transfer. The development of T_FH_ and GCB in the spleen was analysed with FACS 7 days after the cell transfer. Serum levels of anti-BSA-NP antibodies were analysed using enzyme-linked immunosorbent assay (ELISA) 7 days after the immunization, as described above.

### Generation of mixed-BM chimeras

BM cells were harvested by flushing the femurs and tibias of donor mice with RPMI medium. Lethally irradiated Thy1.2^+^ Egr2 CKO mice (700 rad) were reconstituted with 2 × 10^6^ Thy1.2^+^ Egr2 CKO BM cells or a mixture of 1 × 10^6^ Thy1.2^+^ Egr2 CKO and 1 × 10^6^ Thy1.1^+^ WT BM cells. The recipient mice were used in the analyses 6 weeks after BM transfer.

### Localization of splenic Egr2-GFP^+^ T cells

Spleens from Egr2-GFP mice and Foxp3-GFP mice were rapidly frozen in Tissue-Tec OCT compound (Sakura Finetek, Japan) with liquid nitrogen, and then were cut into 5-μm sections with a cryostat microtom. Sections were fixed for 10 min in 4% paraformaldehyde (Wako) and were preincubated in PBS with 2% BSA and 0.1% saponin, and then incubated with the following primary antibodies for 30 min: PE anti-mouse CD4 (5:160), Brilliant Violet 421 anti-mouse B220 (2:160), FITC anti-GFP (3:160). Images were acquired with a fluorescence microscope (Olympus BX53). The frequencies of Egr2-GFP^+^CD4^+^ T cells and Foxp3-GFP^+^CD4^+^ T cells were evaluated by counting the numbers per field; each field was 0.01 mm^2^.

### B-cell isolation and proliferation

Splenic B cells were purified by negative selection with MACS using a B-cell isolation kit (Miltenyi Biotec) according to the manufacturer’s protocol. The purity of MACS-sorted B cells was >95% positive for B220 staining. B cells were labelled with 5 μM 5-(and 6-) carboxyfluorescein diacetate succinimidyl ester (CFSE; Dojindo) at 37 °C for 10 min, and then were stimulated with 10 μg ml^−1^ anti-IgM F(ab)’_2_ (Jackson ImmunoResearch Laboratories) for 72 h with or without rTGF-β1, 2 or 3 for B-cell proliferation assays. Cells were stained with anti-B220 mAb, 7-Amino-Actinomycin D (7-AAD; Biolegend, 3:100) and PE anti-CD40 mAb. The percentages of viable 7-AAD-negative CFSE-diluted B220^+^CD40^+^ B cells and dead 7-AAD-positive B220^+^ cells were assessed using FACS.

### *In vitro* B-cell activation co-culture assays

The wells of 96-well flat-bottomed plates were coated with 10 μg ml^−1^ anti-CD3 mAb in 100 μl per well of PBS and incubated overnight at 4 °C. The wells were washed, and MACS-purified B cells with or without each FACS-purified T-cell subset (LAG3^+^ Treg, CD25^+^ Treg or CD4^+^CD25^−^CD44^lo^CD62L^hi^ naive T cells) or IL-27-treated CD4^+^ T cells described below were plated immediately into the coated wells at a density of 1 × 10^5^ cells per well for each cell type in RPMI medium as described above alone or with 10 μg ml^−1^ anti-CD40 mAb (3/23)+10 μg ml^−1^ rIL-4 (Cell Signaling Technology) supplemented with or without rTGF-β3 (1 ng ml^−1^). B cells undergoing apoptosis on day 3 and total IgG production in the culture supernatants on day 7 were determined using the Annexin V Apoptosis Detection Kit (BD Pharmingen) and a mouse IgG ELISA Quantitation Set (Bethyl Laboratories), respectively, according to the manufacturer’s protocol.

### Adoptive transfer studies in Rag1KO mice and TEa mice

MACS-purified 2 × 10^5^ B cells from B6 or PD-1KO mice and FACS-purified 2 × 10^5^ Th cells from OT-II or PD-1KO OT-II mice were injected i.v. into Rag1KO mice in combination with or without FACS-purified 1 × 10^5^ LAG3^+^ Treg from B6, Egr2 CKO, B6/*lpr*, B6/*gld* or IL-10KO mice, or 3 × 10^5^ IL-27-treated T cells. Control mice received PBS. Mice were subsequently immunized with 100 μg NP-OVA/alum 24 h after the cell transfer. Mice were re-immunized with 50 μg NP-OVA/alum 14 days after the first immunization. Where indicated, the day after the cell transfer, mice were injected i.v. with an anti-FasL blocking antibody (200 μg per mouse), anti-TGF-β1 blocking antibody (100 μg per mouse) or anti-TGF-β3 blocking antibody (100 μg per mouse) at weekly intervals, or an anti-PD-L1 blocking antibody (200 μg per mouse) every 3 days. Serum anti-NP antibody levels were analysed with ELISA, and splenocytes were analysed with FACS 7 days after the re-immunization. To examine the *in vivo* suppressive activity of LAG3^+^ Treg in non-lymphopenic conditions, TEa mice were employed. TEa TCR is supposed to have low crossreactivity^27^ and TEa mice possess very few CD25^+^ Treg^50^. FACS-purified 2 × 10^5^ Th cells from OT-II mice were injected i.v. into TEa mice in combination with or without FACS-purified 1 × 10^5^ LAG3^+^ Treg from B6 mice. Control mice received PBS. Mice were subsequently immunized with 100 μg NP-OVA/alum 24 h after the cell transfer. Serum levels of anti-BSA-NP antibodies were analysed with ELISA 14 days after the immunization, as described above.

### Quantification of NP-specific antibody responses

Anti-NP IgG antibody levels were quantified by ELISA using NP(9)-BSA (Biosearch Technologies) as the capture antigen in the *in vitro* or *in vivo* antibody production assay, respectively. ELISA plates were prepared using the Immuno-Tek ELISA construction system (Zepto Metrix) according to the manufacturer’s protocol. Following the incubation with sample serum or media, the plates were developed with horseradish peroxidase (HRP)-conjugated goat anti-mouse IgG1, or HRP-conjugated goat anti-mouse IgA (SouthernBiotech), and TMB substrate. Serially diluted pooled sera from NP(13)-OVA-immunized B6 mice were included as controls on each plate. The concentrations of the anti-NP IgG1 antibody were estimated by comparisons with standard curves constructed from pooled sera.

### Adoptive transfer studies in MRL/*lpr* mice

Eight-week-old MRL/*lpr* mice were randomly assigned to specific treatment groups. Ten-week-old MRL/*lpr* mice in the treatment group were injected i.v. with LAG3^+^ Treg, LAG3^−^ T cells, CD25^+^ Treg or naive T cells (1 × 10^5^ cells each) obtained from MRL/+ mice. The three-time injection group (MRL/+ LAG3^+^ Treg x3, MRL/+ CD25^+^ Treg x3 and MRL/*lpr* LAG3^+^ Treg x3) was adoptively transferred i.v. with 1 × 10^5^ LAG3^+^ Treg at weekly intervals (10, 11 and 12 or 13, 14 and 15 weeks of age, respectively; the mice were 10 weeks of age at the time of the first injection). Each T-cell subset was first enriched by MACS and then sorted by FACS (on the basis of the expression of CD4, CD25, CD45RB and LAG3) as described above. Mice in the control group received PBS. Where indicated, the day after the first cell transfer, mice were injected i.v. with an anti-TGF-β3 blocking antibody (100 μg per mouse) at weekly intervals. Mice were killed at 18 weeks of age to examine pathological alterations. Anti-ds DNA antibodies were measured using a mouse anti-ds DNA ELISA Kit (Shibayagi) at 13 and 18 weeks of age, according to the manufacturer’s protocol.

### Urinary protein analysis

Proteinuria was assessed semiquantitatively using dip sticks (Albustix, Bayer) at weekly intervals (0=none; 1=30–100 mg dl^−1^; 2=100–300 mg dl^−1^; 3=300–1,000 mg dl^−1^; 4≥1,000 mg dl^−1^).

### Histological analysis

MRL/*lpr* mice were killed at 18 weeks of age. Renal pathology was graded by standard methods for glomerular inflammation, proliferation, crescent formation and necrosis as described elsewhere^49^. The proportion of glomeruli was evaluated by examining at least 80 glomeruli per section by an examiner blind to the experimental conditions. Interstitial and tubular changes were also noted. Scores from 0 to 4 (where 0 represents ‘no damage’ and 4 represents ‘severe’) were assigned for each of these features.

### *In vitro* NP-specific antibody responses

B6 and OT-II mice were immunized with 100 μg NP-OVA/alum. Mice were re-immunized with 100 μg NP-OVA/alum 3 weeks after the first immunization. MACS-purified 2 × 10^5^ B cells from pre-immunized B6 mice and FACS-purified 1 × 10^5^ CD4^+^CD25^−^LAG3^−^ Th cells from pre-immunized OT-II mice were seeded in round-bottom 96-well plates 7 days after the re-immunization in combination with or without FACS-purified 1 × 10^5^ LAG3^+^ Treg from non-immunized OT-II mice in the presence or absence of 10 μg ml^−1^ anti-PD-L1 blocking mAb (10F.9G2) or 10 μg ml^−1^ anti-FasL blocking mAb (MFL3). The culture supernatants were harvested at 3 weeks, and anti-NP antibody levels were analysed with ELISA, as described above.

### RNA isolation, *cDNA synthesis* and quantitative real-time PCR

Total T-cell RNA was prepared using an RNeasy Micro Kit (Qiagen). RNA was reverse-transcribed to cDNA with random primers (Invitrogen) and SuperScript III in accordance with the manufacturer’s protocol (Invitrogen). To determine the cellular expression level of each gene, quantitative real-time PCR analysis was performed using an iCycler (Bio-Rad). The PCR mixture consisted of 25 μl of SYBR Green Master Mix (Qiagen), 15 pmol of forward and reverse primers and the cDNA samples in a total volume of 50 μl (ref. 14). Relative RNA abundance was determined on the basis of control mouse β-actin or human glyceraldehyde-3-phosphate dehydrogenase (GAPDH) abundance.

### DNA microarray analysis

Total RNA of CD4^+^CD25^+^, CD4^+^CD25^−^CD45RB^low^LAG3^+^ and CD4^+^CD25^−^CD45RB^high^LAG3^−^ FACS-purified T cells from B6 mice were harvested and then prepared for Affymetrix microarray analysis as described above. Biotinylated antisense cRNA was prepared using two cycles of *in vitro* amplification according to the Affymetrix Small Sample Labeling Protocol II. Fifteen micrograms of biotinylated cRNA was fragmented and hybridized to Affymetrix GeneChip Mouse Genome 430 2.0 arrays at the Takara Bio Genomics Center. Data were analysed using a Bioconductor (version 1.9) and statistical software R and GeneSpring GX version 7.3.1 (Silicon Genetics). All microarray data have been deposited in the ArrayExpress database ( http://www.ebi.ac.uk/arrayexpress) under accession number E-MEXP-1343. The data set of microarray analysis referred in this article is identical to a data set from our previous study^14^.

### Quantification of TGF-β family members

Each T-cell subset was plated into anti-CD3- or CD3/CD28-coated wells at 3 × 10^5^ cells per well in serum-free X-Vivo-20 medium (Lonza) for the determination of TGF-β1 and 2, or the RPMI medium described above for TGF-β3, respectively. All cultures were incubated at 37 °C for 72 h, and the supernatants were collected and stored at −80 °C before the measurement of TGF-β family members, unless otherwise mentioned. TGF-β1, 2 and 3 levels in supernatants were determined with the TGF-β1 Emax ImmunoAssay System (Promega), TGF-β2 Quantikine ELISA Kit (R&D Systems) and TGF-β3 ELISA Kit (Mybiosource), respectively, according to the manufacturer’s protocol. TGF-β3 levels in the RPMI medium supplemented with 10% FBS were lower than the minimum detectable levels of the TGF-β3 ELISA Kit.

### Western blot analysis

MACS-purified B cells from B6 mice were pretreated with 0.75 μM CpG-ODN (ENZO Life Science) for 72 h supplemented with or without rTGF-β3 (20 ng ml^−1^) during the last 16 h, and subsequently stimulated with 10 μg ml^−1^ anti-CD40 mAb, 10 μg ml^−1^ rIL-4 or 10 μg ml^−1^ anti-IgM F(ab)’_2_ in RPMI medium for the indicated time. Following the stimulation, cells were prepared in Lysis Buffer (50 mM Tris-HCl, 0.15 M NaCl, 1% Triton X-100, 1 mM EDTA), denatured in 2 × Laemmli Buffer (Bio-Rad) at 95 °C for 5 min and resolved on Mini-PROTEAN TGX precast gels (Bio-Rad). Total protein concentrations in the cell lysates were determined using a BCA Protein Assay kit (Pierce). Following blotting on polyvinylidene fluoride membranes and blocking with 5% BSA, blots were probed with antibodies against phospho- or total STAT6, NF-κB p65, or Syk in 1:1,000 dilution (all purchased from Cell Signaling Technology), as well as with secondary anti-rabbit-IgG-HRP (Invitrogen) in 3:10,000 dilution. Membranes were developed with ECL Prime substrate (GE Healthcare). Images have been cropped for presentation. Full-size images are presented in Supplementary Fig. 10

### ChIP sequencing analysis

LAG3^+^ Treg cells were fixed with 1% formaldehyde for 15 min and quenched with 0.125 M glycine. Chromatin was isolated by the addition of lysis buffer, followed by disruption with a Dounce homogenizer. Lysates were sonicated and the DNA sheared to an average length of 300–500 bp. Genomic DNA (Input) was prepared by treating aliquots of chromatin with RNase, proteinase K and heat for de-crosslinking, followed by ethanol precipitation. Pellets were resuspended and the resulting DNA was quantified on a NanoDrop spectrophotometer. Extrapolation to the original chromatin volume allowed quantitation of the total chromatin yield. An aliquot of chromatin (30 μg) was precleared with protein A agarose beads (Invitrogen). Genomic DNA regions of interest were isolated using 4 μg of antibody against Krox20 (Covance, PRB-236P). Complexes were washed, eluted from the beads with SDS buffer and subjected to RNase and proteinase K treatment. Crosslinks were reversed by incubation overnight at 65 °C, and ChIP DNA was purified with phenol–chloroform extraction and ethanol precipitation. Illumina sequencing libraries were prepared from the ChIP and Input DNAs by the standard consecutive enzymatic steps of end-polishing, dA-addition and adaptor ligation. After a final PCR amplification step, the resulting DNA libraries were quantified and sequenced on NextSeq 500. Sequences (75 nt (nucleotides) reads, single end) were aligned to the mouse genome (mm10) using the BWA algorithm (default settings). Duplicate reads were removed and only uniquely mapped reads (mapping quality≥25) were used for further analysis. Alignments were extended *in silico* at their 3′-ends to a length of 200 bp, which is the average genomic fragment length in the size-selected library, and assigned to 32-nt bins along the genome. Krox20 peak locations were determined using the Bioconductor package BayesPeak^51^.

### *In vitro* helper T-cell differentiation and cytokine analysis

MACS-sorted CD4^+^ T cells described above were further purified as CD4^+^CD25^–^CD62L^+^CD44^−^ naive T cells by FACS, and cells were seeded at a density of 3 × 10^5^ cells per 100 μl of RPMI culture medium described above in 96-well plates coated with 2 μg ml^−1^ anti-CD3 mAb and 2 μg ml^−1^ anti-CD28 mAb. Cytokines for effector cell polarization were as follows: Th0, anti-IFN (interferon)-γ (10 μg ml^−1^; XMG1.2) and anti-IL-4 (10 μg ml^−1^; 11B11); Th0, anti-IFN-γ (10 μg ml^−1^; XMG1.2 (BD Pharmingen)) and anti-IL-4 (10 μg ml^−1^; 11B11 (BD Pharmingen)); Th1, IL-12 (10 ng ml^−1^ (R&D Systems)), IL-2 (50 μg ml^−1^ (R&D Systems)) and anti-IL-4 (10 μg ml^−1^; 11B11); Th17, TGF-β1 (1ng ml^−1^), IL-6 (50 ng ml^−1^ (BioLegend)), IL-23 (50 ng ml^−1^ (R&D Systems)), anti-IFN-γ (10 μg ml^−1^; XMG1.2) and anti-IL-4 (10 μg ml^−1^; 11B11); induced Treg, TGF-β1 (5 ng ml^−1^) and anti-IL-4 (10 μg ml^−1^; 11B11). The culture supernatants were harvested on day 5, and TGF-β3 levels were analysed using ELISA, as described above.

### *In vitro* CD4^+^Egr2^+^LAG3^+^ Treg differentiation by IL-27

*In vitro* stimulation of MACS-purified naive CD4^+^ T cells using the CD4^+^CD62L^+^ T Cell Isolation Kit (Miltenyi Biotec) according to the manufacturer’s protocol was performed in 24- or 96-well plates coated with 2 μg ml^−1^ anti-CD3 mAb and 1 μg ml^−1^ anti-CD28 mAb in RPMI medium described above supplemented with 25 ng ml^−1^ IL-27 for 5 days. IL-27-treated naive T cells were subsequently sorted using flow cytometry for CD4 expression and used for each assay. For determination of TGF-β3 levels in the culture supernatants by ELISA, IL-27-treated T cells were stimulated with phorbol myristate acetate (50 ng ml^−1^; Sigma) and ionomycin (1 μg ml^−1^; Sigma) for the last 4 h.

### Construction of the TGF-β3 expression plasmid vector

Full-length fragments of murine TGF-β3 were isolated from an OmicsLink Expression-Ready ORF-cloning vector (GeneCopoeia) containing a *Tgfb3* cDNA (NM_009368). *Tgfb3* cDNAs were subcloned into the pCAGGS vector^52^, which has the CAG (cytomegalovirus immediately early enhancer/chicken β-actin hybrid) promoter, using EcoRI sites and designated as pCAGGs-*Tgfb3*. Recombinant plasmids were then transformed into competent cells of *Escherichia coli* JM109 and purified using plasmid purification columns using the EndFree Plasmid Maxi Kit (Qiagen) according to the manufacturer’s protocol. The purified plasmid DNA was diluted to 1 μg μl^−1^ with sterile PBS (pH 7.4) immediately before use.

### Intravenous injection of plasmid DNA

MRL/*lpr* mice were injected i.v. with 100 μg of plasmid DNA (pCAGGs-*Tgfb3* or control pCAGGS) in sterile PBS (pH 7.4) twice at an interval of 4 weeks. Proteinuria was assessed semiquantitatively at weekly intervals, and renal pathology was evaluated as described above 6 weeks after the final administration.

### Flow cytometric assessment of human PBMCs

All human samples were obtained under informed consent. The protocol for the human research project has been approved by the Ethics Committee of the University of Tokyo. PBMCs from healthy and SLE patients were isolated by Ficoll-Paque (Amersham Pharmacia Biotech) gradient. After the cells were washed, they were stained with indicated the mAbs for 20 min at 4 °C. To prevent nonspecific binding of mAbs, Human Fc Receptor Binding Inhibitor (eBioscience) was added before staining with labelled mAb. Dead cells were excluded by 7-AAD. The fluorescence-positive cells were analysed by a Moflo XDP cell sorter. The five distinct subpopulations are as follows: (naive T) CD4^+^CD25^−^CD45RA^+^CCR7^+^ cells; (CD25^+^ Treg) CD4^+^CD25^+^CD127^dim^CD45RA^−^ cells; (LAG3^+^ Treg) CD4^+^CD25^−^CD45RA^−^LAG3^+^ cells; (Tfh) CD3^+^CD19^−^CD4^+^CD25^−^LAG3^−^CXCR5^+^CD45RA^−^ cells and (B cells) CD3^−^CD19^+^ cells.

### Quantitative real-time PCR expression analysis of human T-cell subsets

Total RNA isolated from human naive T and CD25^+^ Treg, and LAG3^+^ Treg stimulated with plate-bound 5 μg ml^−1^ anti-CD3 for 72 h were analysed for *EGR2, IL10, IFNG*, and *FOXP3* mRNA expressions, as described above. *TGFB3* mRNA expression was determined using unstimulated cells from each T-cell subset.

### Quantification of human IL-10

For cytokine analysis, human naive T, CD25^+^ Treg and LAG3^+^ Treg were plated into 5 μg ml^−1^ anti-CD3/anti-CD28-coated 96-well flat-bottomed plates at 2 × 10^4^ cells per well in RPMI-1640 medium supplemented with 10% FBS, 100 μg ml^−1^ L-glutamine, 100 U ml^−1^ penicillin, 100 μg ml^−1^ streptomycin and 50 μM 2-mercaptoethanol. The culture supernatants were harvested on day 3, and IL-10 levels were analysed with ELISA using OptEIA Human IL-10 ELISA Kit II (BD Biosciences) according to the manufacturer’s protocol.

### Human LAG3^+^ Treg suppression assays

RPMI-1640 medium as described above was used for co-culture. FACS-purified 1 × 10^5^ human B cells and FACS-purified 5 × 10^4^ human T_FH_ cells were seeded in round-bottom 96-well plates with or without FACS-purified 1 × 10^5^ human CD25^+^ Treg or LAG3^+^ Treg in the presence of 2 μg ml^−1^ recombinant staphylococcal enterotoxin B (Toxin Technology). The culture supernatants were harvested on day 12, and total IgG levels were analysed with ELISA using Human IgG Quantitation Set kits (Bethyl Laboratories), according to the manufacturer’s protocol.

### Statistical analysis

Statistical significance, normal distribution and similar variance between groups were analysed using GraphPad Prism version 5.03 (GraphPad Software Inc.). Quantitative histology and proteinuria progression were analysed with the Mann–Whitney *U*-test. For the comparison of more than three groups, a one-way analysis of variance followed by a Bonferroni multiple comparison test was performed. All other statistical differences were determined using the two-tailed Student’s *t*-test. If the variance was unequal, Welch’s correction was applied to Student’s *t*-test. Differences were considered statistically significant at *P*<0.05 for all tests. All data in the figures are expressed as mean±s.d. Sample size was estimated on the basis of numbers typically used in previous studies. No statistical method was used to predetermine sample size. No samples or animals were excluded from the analyses. We did not perform randomization of animals except for adoptive transfer studies in MRL/*lpr* mice. Animal studies were not performed in a blinded manner, except for histological analyses.

## Additional information

**Accession codes:** The accession number for the microarray data presented in this study is E-MEXP-1343 [ArrayExpress database].

**How to cite this article:** Okamura, T. *et al*. TGF-β3-expressing CD4^+^CD25^−^LAG3^+^ regulatory T cells control humoral immune responses. *Nat. Commun.* 6:6329 doi: 10.1038/ncomms7329 (2015).

## Supplementary Material

Supplementary Information Supplementary Figures 1-14 and Supplementary Tables 1-2.
