# Supplementary material for: TGF-β3-expressing CD4+CD25−LAG3+ regulatory T cells control humoral immune responses
Source: Nat Commun. 2015 Feb 19;6:6329. doi: 10.1038/ncomms7329 (PMC4346620; doi:10.1038/ncomms7329)
Supplement: Supplementary Information — Supplementary Figures 1-14 and Supplementary Tables 1-2. [file ncomms7329-s1.pdf]

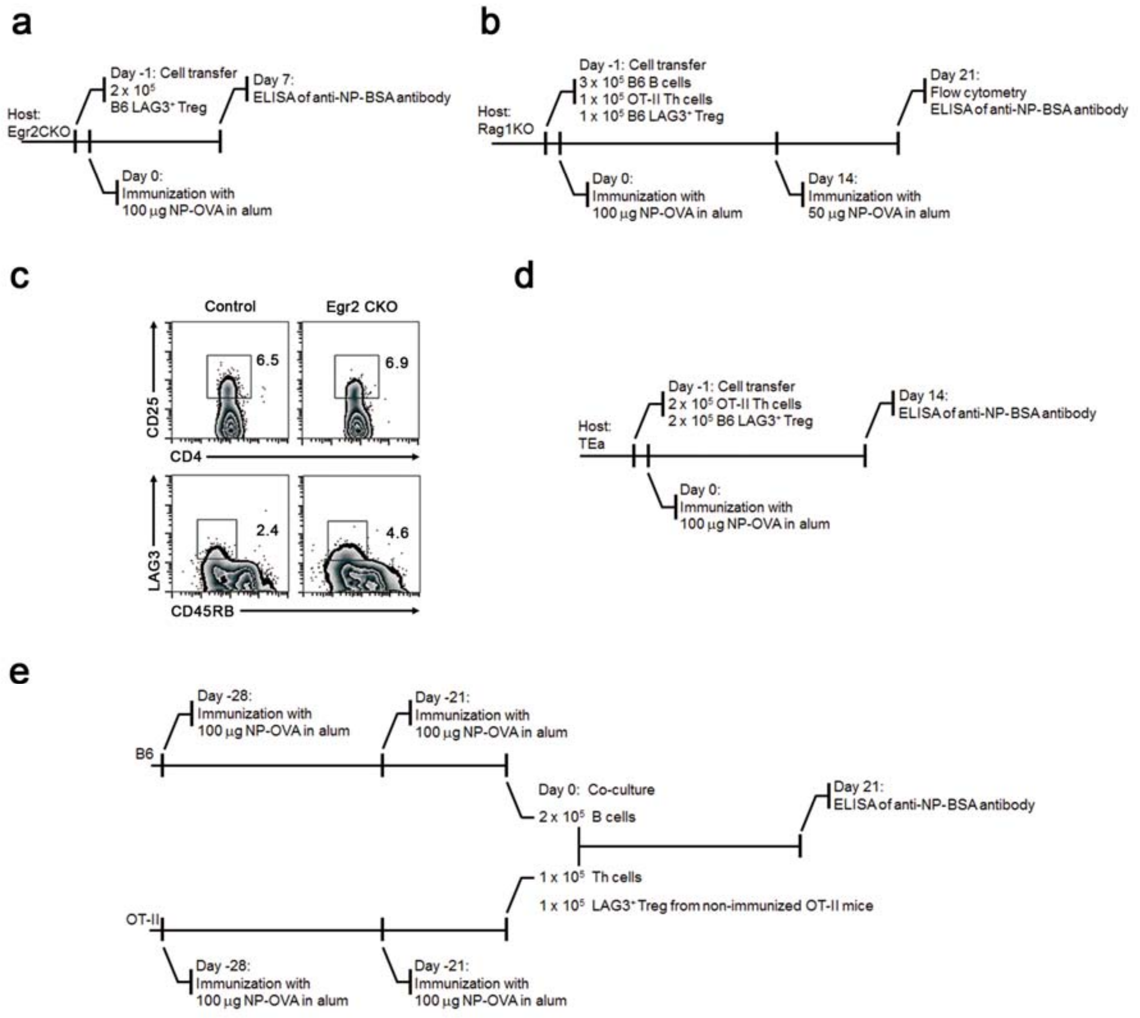

Supplementary Figure 1. LAG3$^+$ Treg-mediated regulation of germinal center B cells and follicular helper T cells is Egr2-dependent. (**a**) Diagrammatic representation of the experimental protocol for the NP-specific antibody responses of WT and *Egr2$^{fl/fl}$* CD4-*Cre*$^+$ (Egr2 CKO) mice immunized once with 100 μg NP-OVA/alum with or without adoptive transfer of WT LAG3$^+$ Treg. The serum levels of anti-NP-BSA antibodies were analyzed by ELISA 7 d after immunization. (**b**) Diagrammatic representation of the experimental

protocol for cell transfer into Rag1-deficient (Rag1KO) mice immunized twice with NP-OVA/alum. C57BL/6 (B6) B cells and OT-II $CD4^+CD25^-LAG3^-$ helper T (Th) cells were injected *i.v.* into Rag1KO mice in combination with or without $LAG3^+$ Treg from B6 mice 1 d before the *i.p.* immunization with NP-OVA/alum, and given a booster immunization 14 d after the primary immunization. Anti-NP-BSA antibodies in sera were analyzed by ELISA 7 d after the booster immunization. (**c**) Splenic $LAG3^+$ Treg in Egr2 CKO mice. Dot plots were gated on $CD4^+$ T cells (upper panels) and $CD4^+CD25^-$ T cells (lower panels). Numbers indicate the percentage of cells contained within the rectangular regions. The data shown are representative of four independent experiments. (**d**) Diagrammatic representation of the experimental protocol for $LAG3^+$ Treg transfer into TEa TCR transgenic mice. $LAG3^+$ Treg from B6 mice and OT-II Th cells were injected *i.v.* into TEa mice and subsequently immunized with NP-OVA/alum once. Anti-NP-BSA antibody levels were determined by ELISA. (**e**) *In vitro* NP-specific antibody production. B cells and Th cells purified from NP-OVA/alum-pre-immunized B6 mice and OT-II mice, respectively, were incubated with or without $LAG3^+$ Treg from non-immunized OT-II mice in the presence or absence of anti-FasL blocking antibody, and supernatants were analyzed for anti-NP-BSA antibodies by ELISA.

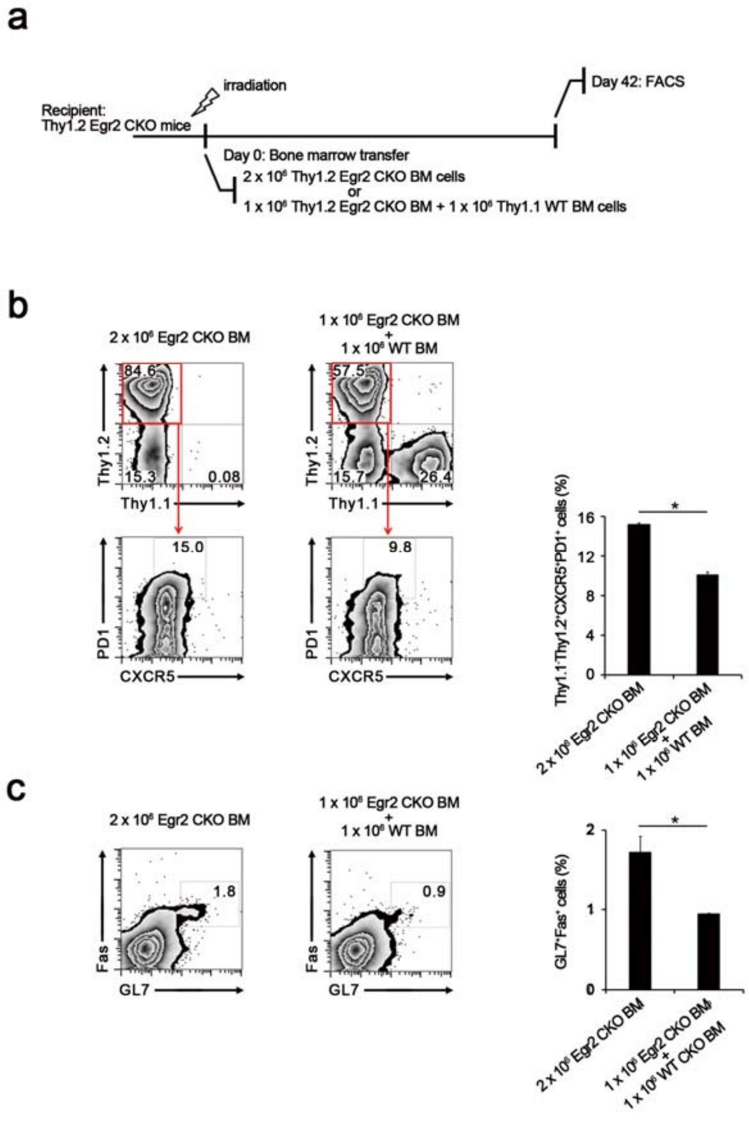

Supplementary Figure 2. Bone marrow chimeras of Egr2 CKO mice reconstituted with wild-type and/or Egr2 CKO bone marrow cells (**a**) Diagrammatic representation of the experimental protocol for the bone marrow (BM) transfer experiment. Lethally irradiated Thy1.2^{+} Egr2 CKO recipients were reconstituted with 2 x 10^{6} Thy1.2^{+} Egr2 CKO BM cells or a mixture of 1 x 10^{6} Thy1.2^{+} Egr2 CKO and 1 x 10^{6} Thy1.1^{+} wild-type (WT)

BM cells. (**b, c**) Flow cytometry plots and quantification of $CD4^+CD25^-CXCR5^+PD\text{-}1^+$ T_{FH} (b) and $B220^+GL\text{-}7^+Fas^+$ GCB (c) 6 weeks after BM transfer (*n* = 4 per group). Percent chimerism was determined in the spleen of the recipient mice by staining for Thy1.1 and Thy1.2 (left upper panels) (b). *$P < 0.05$ (unpaired two-tailed Student's *t*-test). Data are representative of three independent experiments. The means ± s.d. are indicated.

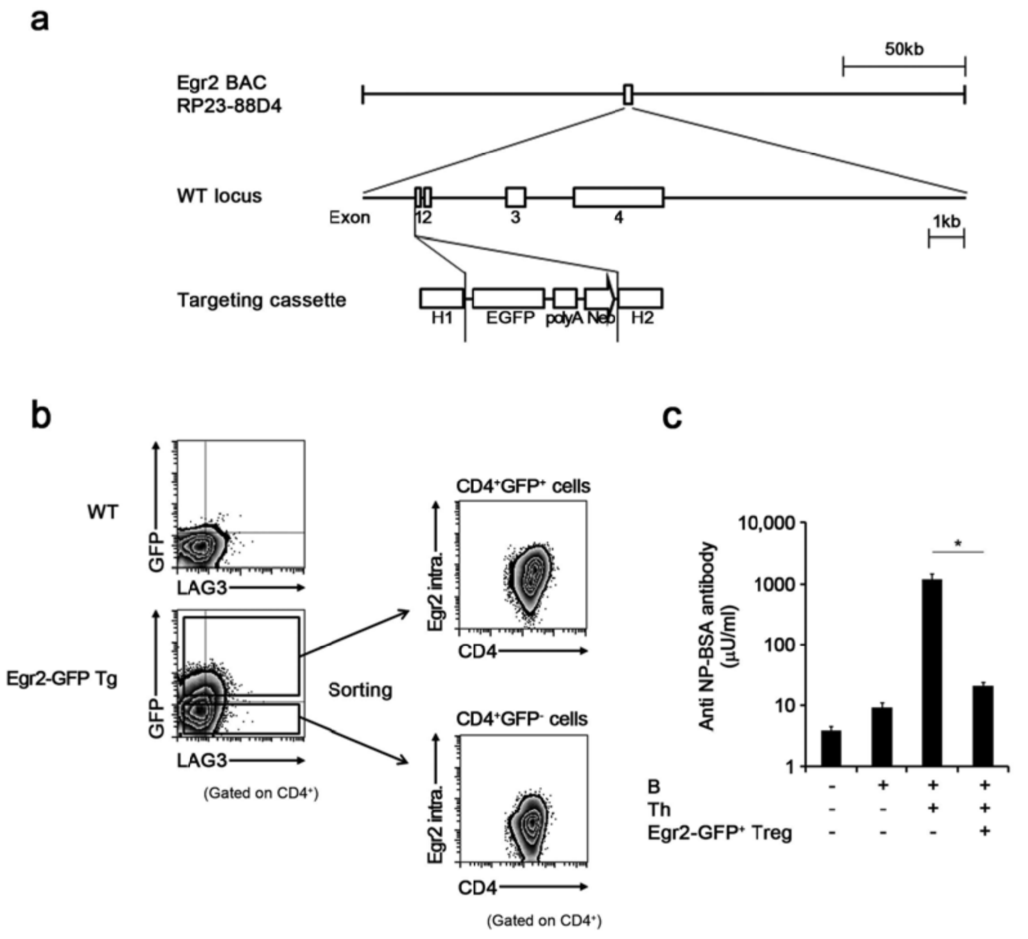

Supplementary Figure 3. Regulatory activity of CD4$^+$CD25$^-$Egr2-GFP$^+$ T cells from Egr2-GFP transgenic mice. (**a**) A schematic representation of the targeting strategy using an Egr2 BAC clone (RP23-884D) to produce Egr2-GFP transgenic mice (Egr2-GFP mice). A part of the *Egr2* coding region was replaced by the EGFP-SV40 poly A cassette. H1 and H2, homology arms; Neo, neomycin-resistant gene. (**b**) GFP expression in Egr2-GFP mice. The sorting strategy of CD4$^+$ cells into CD4$^+$GFP$^+$ and CD4$^+$GFP$^-$ cells from Egr2-GFP mice is shown in the lower left panel. Re-sorted T cells were stained intracellularly with an

anti-Egr2 monoclonal antibody (mAb). Data are representative of three independent experiments. (**c**) $CD4^+CD25^-Egr2\text{-}GFP^+$ T cell-mediated *in vivo* B cell suppression. NP-specific antibody responses of Rag1KO mice injected with B6 B cells and OT-II Th cells in combination with or without $CD4^+CD25^-Egr2\text{-}GFP^+$ T cells (Egr2-GFP^+ Treg) from Egr2-GFP mice (n = 6 per group). Anti-NP antibody levels were determined as in **Fig. 1e**. Statistical analyses were performed using one-way analysis of variance (ANOVA) with Bonferroni post-test (*$P < 0.05$). The means ± s.d. are indicated.

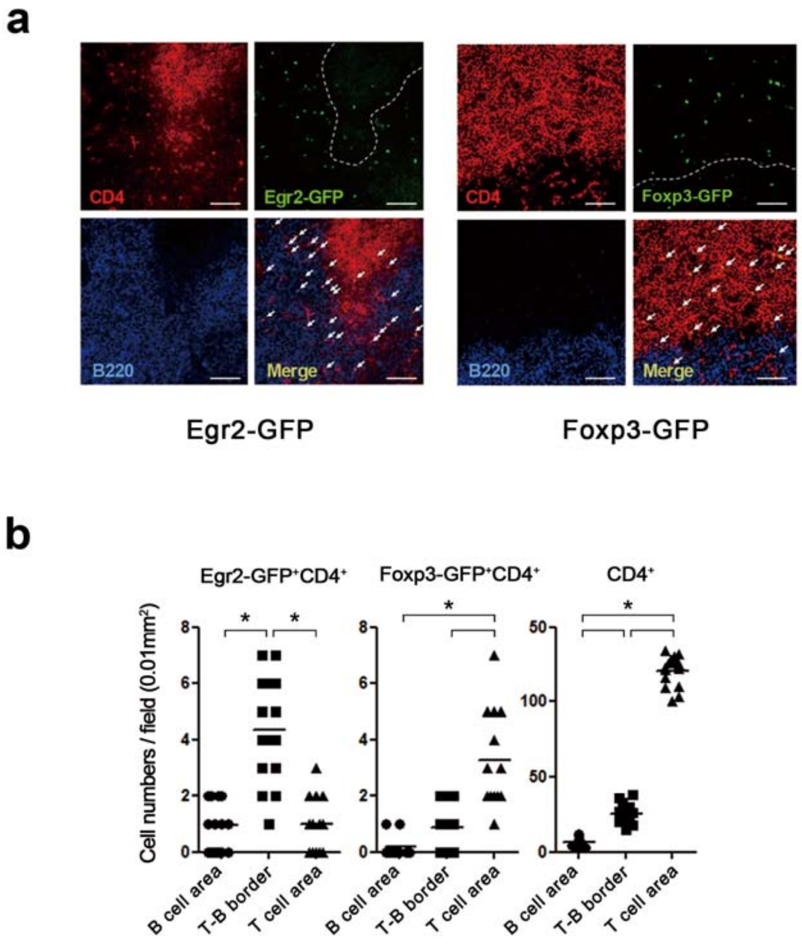

Supplementary Figure 4. Localization of Egr2$^+$CD4$^+$ T cells and Foxp3$^+$CD4$^+$ T cells. (**a**) Representative immunofluorescence staining of frozen sections of splenic tissue harvested from Egr2-GFP (left panels) and Foxp3-GFP mice (right panels). Tissue sections were stained for CD4 (red), B220 (blue), and GFP (green). White arrows indicate CD4$^+$ T cells expressing Egr2-GFP or Foxp3-GFP. (**b**) Frequencies of Egr2-GFP$^+$CD4$^+$ T cells and Foxp3-GFP$^+$CD4$^+$ T cells (cell numbers per field; each field 0.01 mm^2). Statistical analyses were performed using one-way ANOVA with Bonferroni post-test (*$P < 0.05$).The means ± s.d. are indicated.

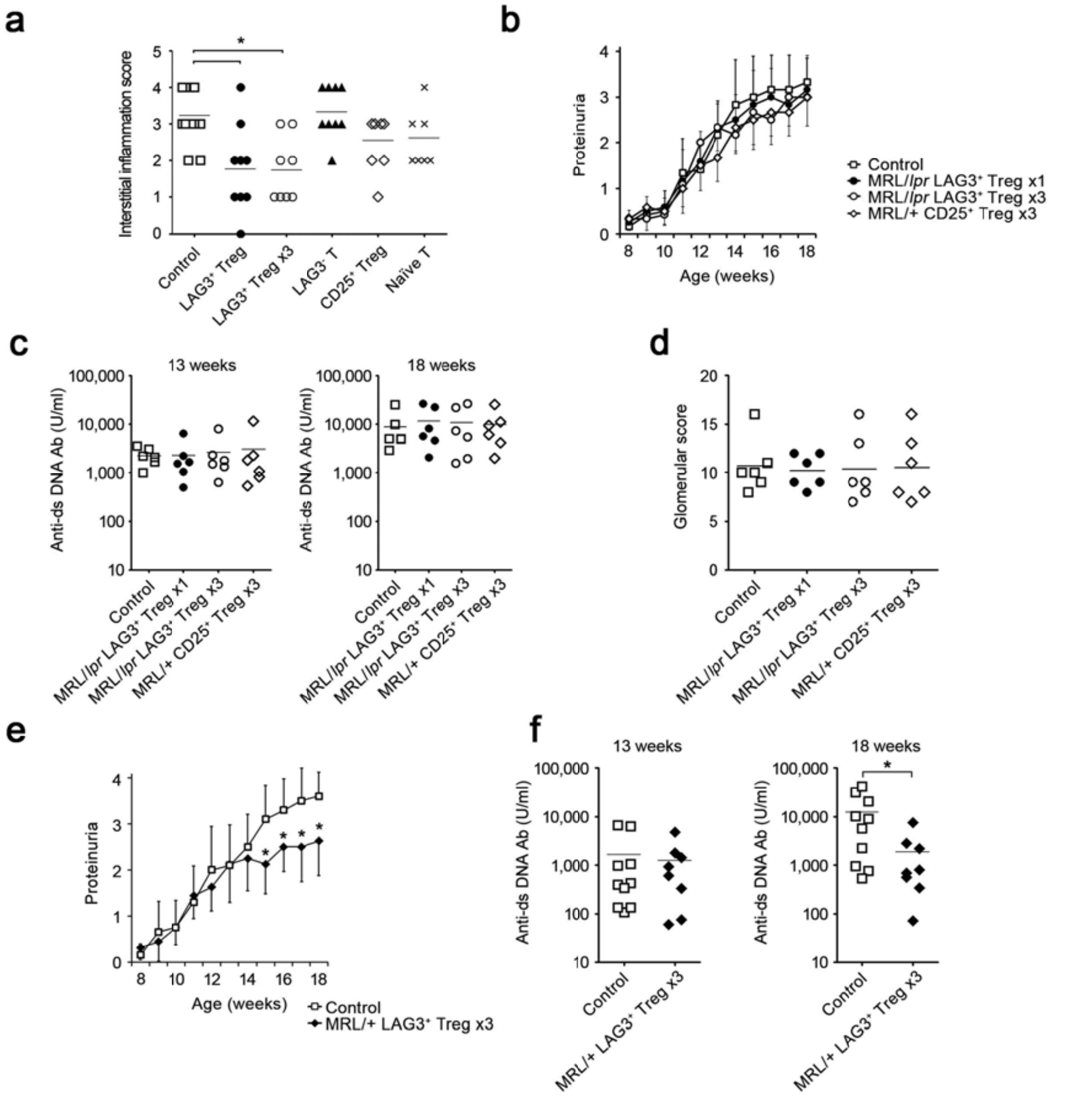

Supplementary Figure 5. LAG3$^+$ Treg ameliorate systemic lupus erythematosus in the murine model. (**a**) Interstitial inflammation scores of kidney sections. At 10 weeks of age, MRL/*lpr* mice in the treatment group were injected *i.v.* with CD4$^+$CD25$^-$LAG3$^+$ Treg (LAG3$^+$ Treg) (n = 9), CD4$^+$CD25$^-$LAG3$^-$ T cells (LAG3$^-$ T) (n = 9), CD4$^+$CD25$^+$ Treg (CD25$^+$ Treg) (n = 9), or CD4$^+$CD25$^-$CD45RB^{high} T cells (naïve T) (n = 8) from MRL/+ mice (1 x 10^5 cells each). In one group (LAG3$^+$ Treg x3) (n = 8), mice were first injected

with LAG3$^+$ Treg (1 x 10^5 cells) at 10 weeks of age followed by a twice-weekly injection. Mice in the control group received PBS (*n* = 13). The data source is identical to **Fig. 2a**-**d**. Interstitial inflammation in each kidney section was graded by standard methods, as described in **Methods**. *$P < 0.05$ (Mann-Whitney *U*-test). (**b**-**d**) MRL/*lpr* mice treated with LAG3$^+$ Treg from MRL/*lpr* mice or CD25$^+$ Treg from MRL/+ mice (*n* = 6 per group). All other conditions were identical to **Supplementary Figure 5a**, except that LAG3$^+$ Treg from MRL/*lpr* mice (x1 and x3) and CD25$^+$ Treg from MRL/+ mice (x3) were used. *n* = 6 mice per group. The levels of proteinuria (**b**) *$P < 0.05$ *vs* control group (Mann-Whitney *U*-test), anti-ds DNA antibodies (**c**) *$P < 0.05$ (Bonferroni post-test), and glomerular scores (**d**) *$P < 0.05$ (Mann-Whitney *U*-test) were examined using the same methods as in **Fig. 2a**-**d**. (**e,f**) Three time injections of LAG3$^+$ Treg after the onset of overt proteinuria. MRL/*lpr* mice were first injected with LAG3$^+$ Treg from MRL/+ mice (1 x 10^5 cells) at 13 weeks of age followed by a twice weekly injection (MRL/+ LAG3$^+$ Treg x3). Mice in the control group received PBS. The levels of proteinuria (**e**) and anti-ds DNA antibodies (**f**) were examined using the same methods as in **Fig. 2a,b** (Control, *n* = 10; LAG3$^+$ Treg x3, *n* = 8). Statistical significances in **e** were analyzed by Mann-Whitney *U*-test, and **f** were analyzed by unpaired two-tailed Student's *t*-test (*$P < 0.05$). The means ± s.d. are indicated.

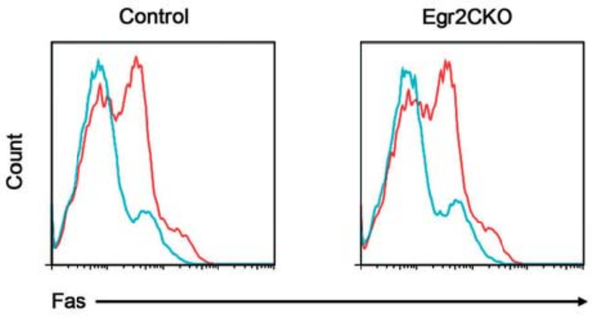

Supplementary Figure 6. Expression of Fas in Egr2 CKO mice. Freshly isolated splenic $CD4^+$ T cells from WT (*Egr2*$^{fl/fl}$ CD4-*Cre*$^-$, left panel) and Egr2 CKO mice (*Egr2*$^{fl/fl}$ CD4-*Cre*$^+$, right panel) were incubated in anti-CD3 mAb-coated 96-well culture plates at 1 x 10^5 cells per well for 72 h. Representative histograms show LAG3 positive (red line) or LAG3 negative cells (blue line). Histograms are gated on $CD4^+$ T cells. Representative data from three independent experiments are shown.

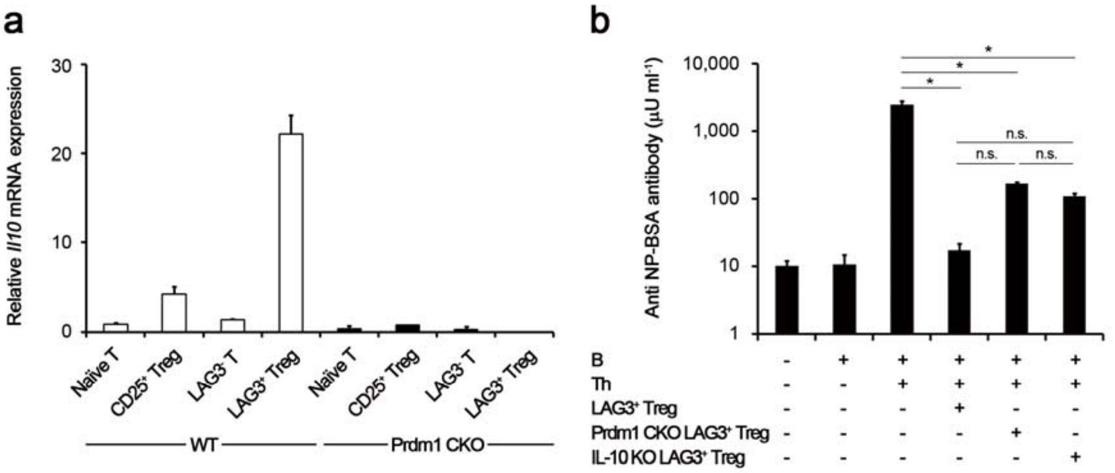

Supplementary Figure 7. LAG3^{+} Treg in Prdm1 CKO and IL-10KO mice. (**a**) *Il10* mRNA expression by sorted T cell subsets taken from the spleens of WT B6 or Prdm1 CKO mice (*Prdm1$^{fl/fl}$* CD4-*Cre*$^{-}$). The four distinct T cell subsets are as follows: (naïve T) CD4^{+}CD25^{-}LAG3^{-}CD45RB^{high} cells; (CD25^{+} Treg) CD4^{+}CD25^{+}LAG3^{-} cells; (LAG3^{-} T) CD4^{+}CD25^{-}LAG3^{-} T cells; (LAG3^{+} Treg) CD4^{+}CD25^{-}LAG3^{+} cells (*n* = 3 per group). (**b**) NP-specific antibody responses of Rag1 KO mice injected with B6 B cells and OT-II Th cells in combination with or without LAG3^{+} Treg from WT B6, Prdm1 CKO or IL-10 KO mice (*n* = 5 per group). Anti-NP-BSA antibody levels were determined as in **Fig. 1e**. Statistical analyses were performed using one-way ANOVA with Bonferroni post-test (*P < 0.05) ; n.s., not significant. See also **Supplementary Tables 1** and **2**. Data are representative of three independent experiments. The means ± s.d. are indicated.

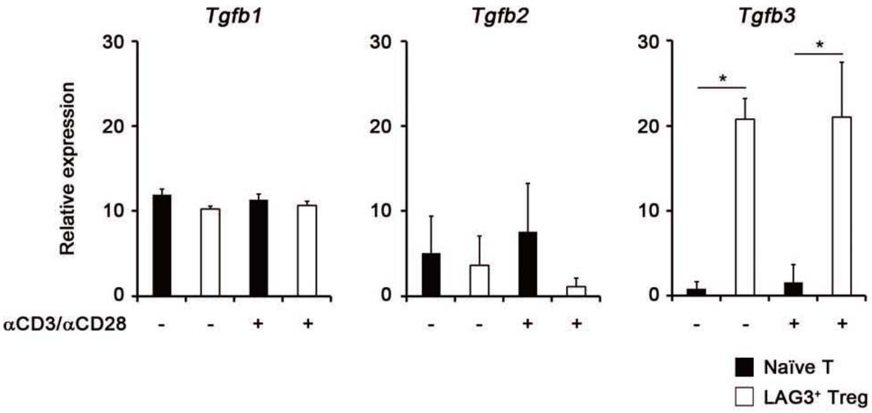

Supplementary Figure 8. Expression of *Tgfb1, Tgfb2, and Tgfb3* mRNA in naïve T cells and LAG3$^+$ Treg. Freshly isolated splenic naïve CD4$^+$ T cells and LAG3$^+$ Treg from B6 mice (n = 3 per group) were stimulated with or without plate-bound 10 μg ml^{-1} anti-CD3/ 5 μg ml^{-1} anti-CD28 antibody for 48 h in RPMI-1640 medium supplemented with 10% FBS, and total RNA extracted from each subset was analyzed for *Tgfb1, Tgfb2, and Tgfb3* mRNA expressions. Statistical analyses were performed using unpaired two-tailed Student's t-test (*P < 0.05). Data are representative of three independent experiments. The means ± s.d. are indicated.

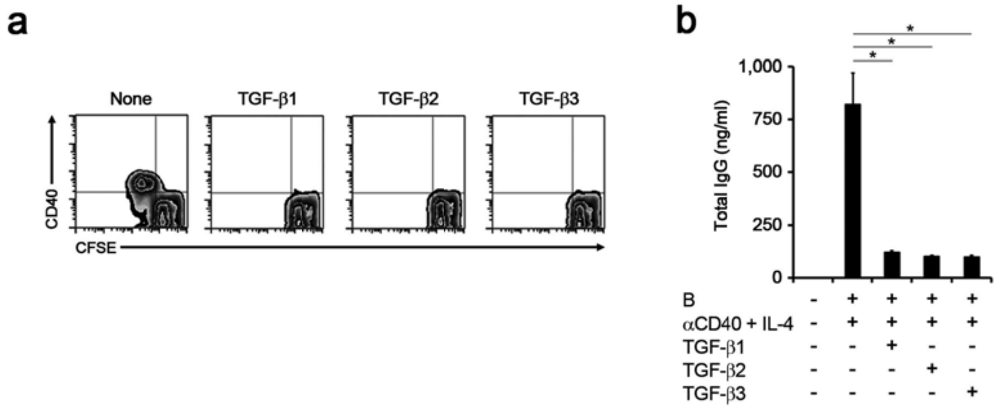

Supplementary Figure 9. Suppression of B cell activation mediated by TGF-β1, 2, and 3. (**a**) CFSE-labeled splenic B cells from naïve B6 mice were stimulated *in vitro* for 72 h with anti-IgM mAb in the presence or absence of rTGF-β1 (1 ng ml^{-1}), rTGF- β2 (1 ng ml^{-1}), or rTGF- β3 (1 ng ml^{-1}). Dot plots are gated on $B220^{+}$ B cells. (**b**) The effects of rTGF-β1, 2, or 3 on total IgG production in the culture supernatants of anti-CD40/IL-4-stimulated B cells. Total IgG levels were determined as in **Fig. 1d** ($n = 3$ per group). Data are representative of three independent experiments ($n \geq 3$ mice per group). Statistical analyses were performed using one-way analysis of variance (ANOVA) with Bonferroni post-test (*$P < 0.05$). Data are representative of three independent experiments. The means ± s.d. are indicated.

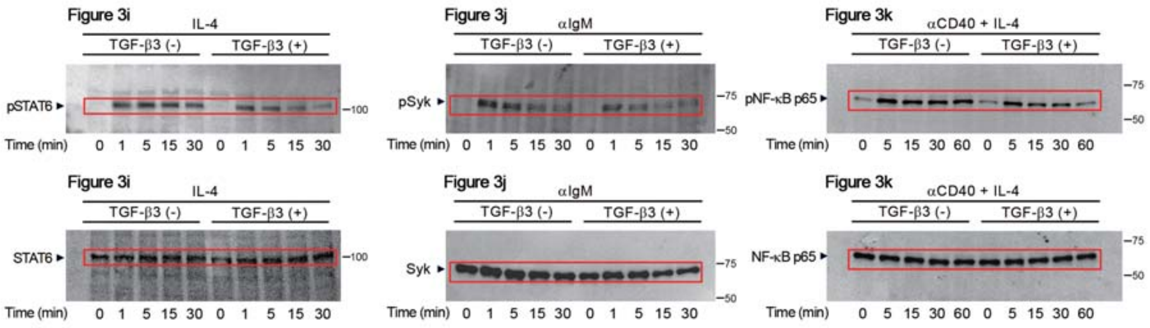

Supplementary Figure 10. Full immunoblots with indicated areas of selection.

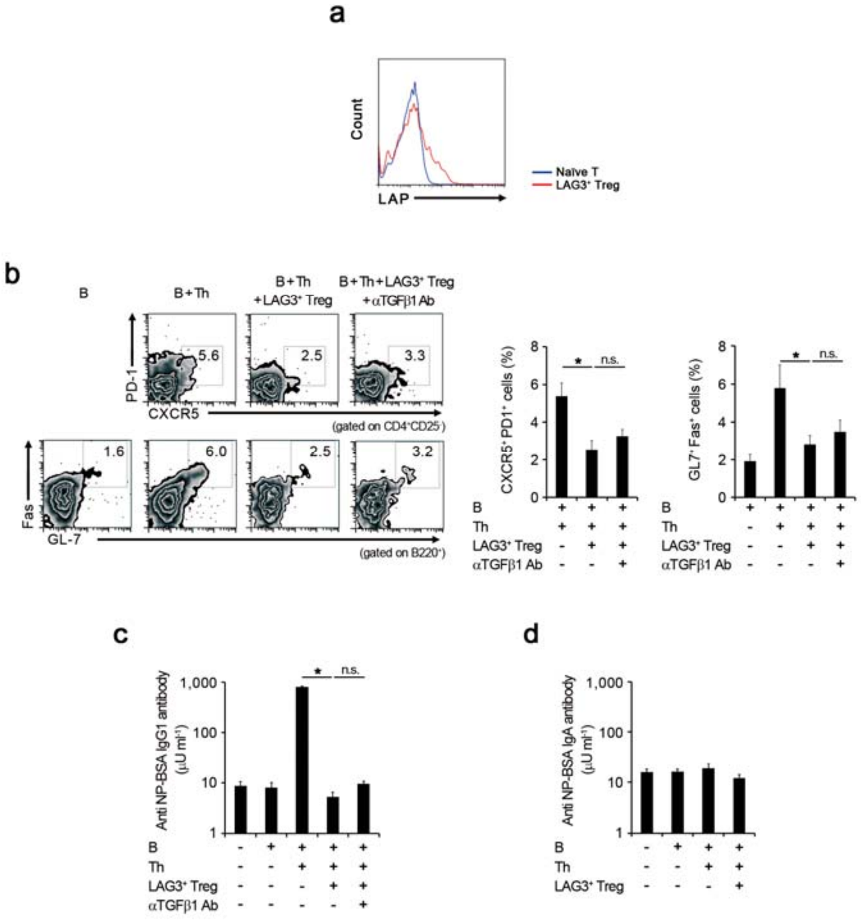

Supplementary Figure 11. TGF-β1-independent suppression of humoral responses by LAG3$^+$ Treg. (**a**) Expression of LAP-TGF-β1 on LAG3$^+$ Treg. Freshly isolated splenic naïve T cells and LAG3$^+$ Treg from B6 mice were stimulated with plate-bound anti-CD3/anti-CD28 antibody for 72 h in RPMI-1640 medium supplemented with 10% FBS. Representative histograms show naïve T cells (blue line) or LAG3$^+$ Treg (red line). Histograms are gated on CD4$^+$ T cells. (**b**) Flow cytometry plots and quantification of splenic CD4$^+$CD25$^-$CXCR5$^+$PD1$^+$ T_{FH} and B220$^+$GL7$^+$Fas$^+$ GCB. B6 B cells and OT-II Th cells were transferred with or without B6 LAG3$^+$ Treg into Rag1KO mice immunized twice with NP-OVA/alum, as described in **Supplementary Fig. 1b** (*n* = 6 per group). Anti-TGF-β1 blocking antibody (100 μg per mouse) was injected *i.v.* weekly. Numbers

indicate the percentage of cells contained within the rectangular regions. (**c**) Serum levels of anti-NP-specific IgG1 antibody from the same mice as in **b**. (**d**) Serum levels of anti-NP-specific IgA antibody from the same mice as in **b**. $^*P < 0.05$ as calculated by one-way ANOVA with Bonferroni post-test. The means ± s.d. are indicated.

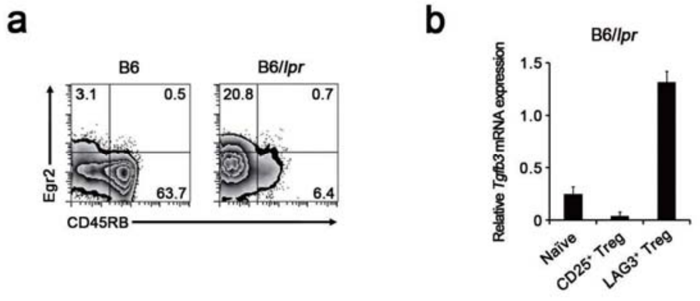

Supplementary Figure 12. Fas-independent expression of *Tgfb3* mRNA in LAG3$^+$ Treg. (**a**) Splenic LAG3$^+$ Treg in B6 and B6/*lpr* mice. Dot plots were gated on CD4$^+$CD25$^-$ T cells. Numbers indicate the percentage of cells contained within the quadrant regions. (**b**) Freshly isolated splenic naïve T cells, CD25$^+$ Treg, and LAG3$^+$ Treg from B6/*lpr* mice (*n* = 3 per group) were stimulated with plate-bound anti-CD3/anti-CD28 antibody for 48 h in RPMI-1640 medium supplemented with 10% FBS, and total RNA extracted from each subset was analyzed for *Tgfb3* mRNA expressions. Data are representative of three independent experiments. The means ± s.d. are indicated.

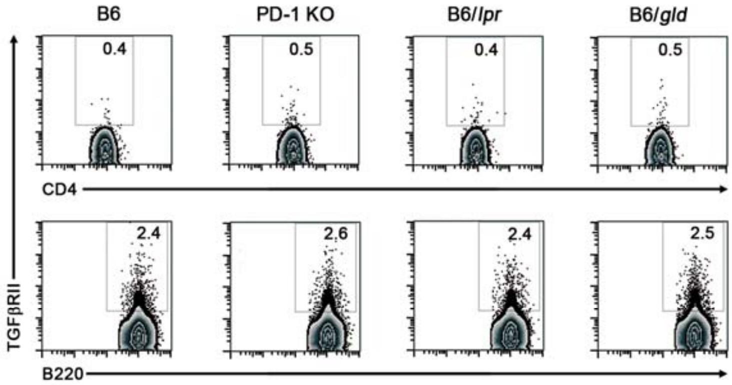

Supplementary Figure 13. Expression of TGFbRII on T cells and B cells. Freshly isolated splenocytes from B6, PD-1 KO, B6/*lpr*, and B6/*gld* mice were analyzed for the expression of TGFβRII. Dot plots were gated on $CD4^+$ T cells (upper panels) and $B220^+$ B cells (lower panels). Numbers indicate the percentage of cells contained within the rectangular regions. The data shown are representative of three independent experiments.

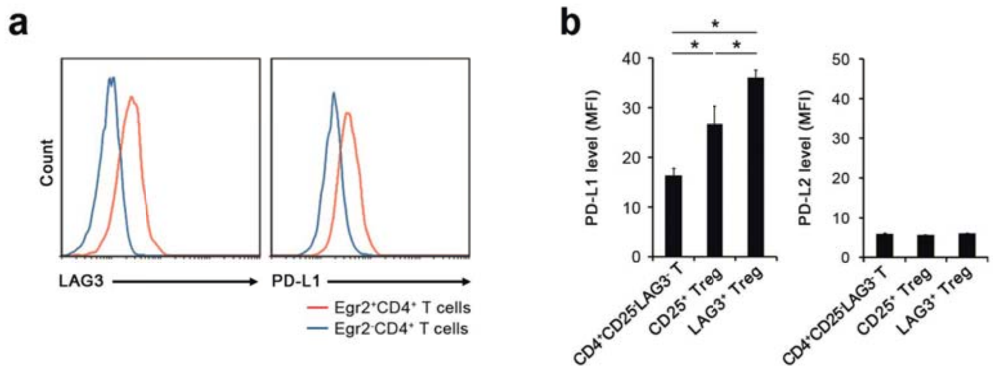

Supplementary Figure 14. LAG3$^+$ Treg express PD-L1. (**a**) Co-expression of LAG3 and PD-L1 on CD4$^+$CD25$^-$Egr2$^+$ T cells from the spleens of B6 mice. Freshly isolated splenic CD4$^+$CD25$^-$ T cells were analyzed for the expression of LAG3 (left panel) and PD-L1 (right panel) according to their intracellular Egr2 expression. (**b**) Freshly isolated splenocytes from B6 mice were stained with anti-CD4 mAb, anti-CD25 mAb, anti-LAG3 mAb, and PD-L1 mAb or PD-L2 mAb. The mean fluorescent intensity (MFI) levels of PD-L1 (left panel) and PD-L2 (right panel) in CD4$^+$CD25$^-$LAG3$^-$ T cells, CD25$^+$ Treg, and LAG3$^+$ Treg are shown (n = 4 per group). *$P < 0.05$ as calculated by one-way ANOVA with Bonferroni post-test. Data are representative of three independent experiments. The means ± s.d. are indicated.

Supplementary Table 1. Mean and standard deviation of anti NP-BSA antibody titers in Supplementary Fig. 7

	None	B	B + Th	B + Th + LAG3$^+$ Treg	B + Th + Prdm1CKO LAG3$^+$ Treg	B + Th + IL10KO LAG3$^+$ Treg
**average**	9.83	11.04	2424.80	16.74	171.77	107.80
**s.d.**	1.44	3.06	268.77	3.40	5.08	9.97

(μU ml^{-1})

Supplementary Table 2. Boferroni adjusted *P* values for multiple comparisons of anti NP-BSA antibody titers in Supplementary Fig. 7

	Adjusted *P* Value	Summary
**None vs B only**	> 0.9999	n.s.
**None vs B + Th**	< 0.0001	*
**None vs B + Th + LAG3$^+$ Treg**	> 0.9999	n.s.
**None vs B + Th + Prdm1CKO LAG3$^+$ Treg**	0.4271	n.s.
**None vs B + Th + IL10KO LAG3$^+$ Treg**	> 0.9999	n.s.
**B only vs B + Th**	< 0.0001	*
**B only vs B + Th + LAG3$^+$ Treg**	> 0.9999	n.s.
**B only vs B + Th + Prdm1CKO LAG3$^+$ Treg**	0.4434	n.s.
**B only vs B + Th + IL10KO LAG3$^+$ Treg**	> 0.9999	n.s.
**B + Th vs B + Th + LAG3$^+$ Treg**	< 0.0001	*
**B + Th vs B + Th + Prdm1CKO LAG3$^+$ Treg**	< 0.0001	*
**B + Th vs B + Th + IL10KO LAG3$^+$ Treg**	< 0.0001	*
**B + Th + LAG3$^+$ Treg vs B + Th + Prdm1CKO LAG3$^+$ Treg**	0.5285	n.s.
**B + Th + LAG3$^+$ Treg vs B + Th + IL10KO LAG3$^+$ Treg**	> 0.9999	n.s.
**B + Th + Prdm1CKO LAG3$^+$ Treg vs B + Th + IL10KO LAG3$^+$ Treg**	> 0.9999	n.s.

Statistical analyses were performed using one-way ANOVA with Bonferroni post-test (*P < 0.05) ; n.s., not significant.
